# Platelet-derived extracellular vesicles induced through different activation pathways drive melanoma progression by functional and transcriptional changes

**DOI:** 10.1186/s12964-024-01973-4

**Published:** 2024-12-18

**Authors:** Zeynep Tavukcuoglu, Umar Butt, Alessandra V. S. Faria, Johannes Oesterreicher, Wolfgang Holnthoner, Saara Laitinen, Mari Palviainen, Pia R-M Siljander

**Affiliations:** 1 EV group, Molecular and Integrative Biosciences Research Programme, Faculty of Biological and Environmental Sciences, and CURED, Drug Research Program, Faculty of Pharmacy, Division of Pharmaceutical Biosciences, University of Helsinki, Viikinkaari 9, Helsinki, 00790 Finland; 2 Faculdade Israelita de Ciências da Saúde Albert Einstein, Hospital Israelita Albert Einstein, São Paulo, SP Brazil; 3 AUVA Research Centre, Ludwig Boltzmann Institute for Traumatology, Vienna, Austria; 4 Finnish Red Cross Blood Service (FRCBS), Helsinki, Finland; 5 EV Core, Molecular and Integrative Biosciences Research Programme, Faculty of Biological and Environmental Sciences, University of Helsinki, Helsinki, Finland

**Keywords:** Platelet, Extracellular vesicles, Melanoma, Functional assays, RNA sequencing, 3D cell culture

## Abstract

**Background:**

Beyond their conventional roles in hemostasis and wound healing, platelets have been shown to facilitate hematogenous metastasis by interacting with cancer cells. Depending on the activation route, platelets also generate different platelet-derived extracellular vesicles (PEVs) that may educate cancer cells in the circulation or within the tumor microenvironment. We engaged different platelet-activating receptors, including glycoprotein VI and C-type lectin-like receptor 2, to generate a spectrum of PEV types. This allowed us to investigate the differential capacity of PEVs to alter cancer hallmark functions such as proliferation, invasion, and pro-angiogenic potential using melanoma as a model. Additionally, we analyzed changes in the cell transcriptomes and cancer EV profiles.

**Methods:**

Two human melanoma cell lines (MV3 and A2058) with differential metastatic potential were studied in the 3D spheroid cultures. Human platelets were activated with collagen related peptide (CRP), fucoidan from *Fucus vesiculosus* (FFV), thrombin & collagen co-stimulus and Ca^2+^ ionophore, and PEVs were isolated by size-exclusion chromatography followed by ultrafiltration. Spheroids or cells were treated with PEVs and used in functional assays of proliferation, invasion, and endothelial tube formation as well as for the analysis of cancer EV production and their tetraspanin profiles. Differentially expressed genes and enriched signaling pathways in the PEV-treated spheroids were analyzed at 6 h and 24 h by RNA sequencing.

**Results:**

Among the studied PEVs, those generated by CRP and FFV exhibited the most pronounced effects on altering cancer hallmark functions. Specifically, CRP and FFV PEVs increased proliferation in both MV3 and A2058 spheroids. Distinct tetraspanin signatures of melanoma EVs were induced by all PEV types. While the PI3K-Akt and MAPK signaling pathways were activated by both CRP and FFV PEVs, they differently upregulated the immunomodulatory TGF-β and type-I interferon signaling pathways, respectively.

**Conclusions:**

Our study revealed both shared and distinct, cancer-promoting functions of PEVs, which contributed to the transcriptome and metastatic capabilities of the melanoma spheroids. Inhibiting the platelet receptors that modulate the PEVs’ cancer-promoting properties may open up new strategies for identifying promising treatment targets for cancer therapy.

**Supplementary Information:**

The online version contains supplementary material available at 10.1186/s12964-024-01973-4.

## Background

Extracellular vesicles (EVs) are lipid bilayer-enclosed particles released by all cells constitutively or activation-dependently [1]. EVs mediate intercellular communication by transporting a variety of bioactive molecules, including proteins, lipids, and nucleic acids, between cells at the primary tumor site and in circulation [2]. Circulating EVs are derived from all blood cells, platelets being among the most abundant cell source [3]. In addition to their established roles in haemostasis and thrombosis, platelets have been shown to contribute to cancer progression and metastasis [4]. In blood, platelets may interact with circulating tumor cells (CTCs), where CTCs aggregate and/or activate platelets either via direct receptor-ligand interactions or soluble cytokines. Such platelet-cancer cell molecular pairs include glycoprotein VI (GPVI) and galectin 3 and C-type lectin 2 receptor (CLEC-2) and podoplanin. Further, platelets protect CTCs from destruction by high shear forces and immune surveillance by forming a physical cloak and subsequently guiding the formation of early metastatic niches [4, 5]. Platelets have been shown to aid CTC endothelial adherence and extravasation into the tumor microenvironment [6]. Although so far there is less evidence for it, platelets have been reported to also infiltrate solid tumors in vivo, transferring their bioactive cargo (e.g. mRNAs, microRNAs) to cancer cells [7, 8].

Upon activation, platelets release a heterogeneous population of activation-tunable platelet-derived extracellular vesicles (PEVs) [9] which may provide an additional means of modulating hematogenous metastasis, and to facilitate pre-metastatic niche formation [8, 10]. PEVs have previously been reported to contribute to cancer hallmarks, including invasiveness [11], migration [12, 13], angiogenesis [14], and evasion of the immune response [15] in colon, breast, and lung cancers. The majority of previous studies have highlighted PEVs as cancer-promoting agents, but a few studies have also suggested their cancer-suppressive roles [7, 16]. Finally, PEVs might enhance the cancer cell resistance to drug-induced cell death by altering cell metabolism [17–19]. However, currently there is more evidence to support beneficial utilization of PEVs as drug delivery vehicles for loading anti-cancer drugs in therapeutic applications [20–22].

GPVI and CLEC-2 are important immunomodulatory platelet receptors that share a common signaling pathway downstream of a hemi-immunoreceptor tyrosine-based activation motif, analogous to that in leukocytes [23]. In vivo findings from syngeneic models of metastasis indicate that GPVI promotes metastasis in melanoma and lung cancer, and inhibition of GPVI offers a completely new therapeutic approach with minimal bleeding [24, 25]. Podoplanin, a transmembrane protein that binds to the CLEC-2 receptor, is upregulated in melanoma cells, and this interaction has been shown to boost platelet-tumor cell aggregation, aiding tumor cell survival and metastasis [26]. To study the mechanisms by which platelets via differently tuned PEVs may impact melanoma, we activated platelets through GPVI using collagen related peptide (CRP) and through all thrombin and collagen receptors including GPVI using thrombin and collagen co-stimulus [27]. To engage CLEC-2, we used fucoidan from *Fucus vesiculosus* (FFV) [28], although it was recently shown that FFV also likely engages platelet endothelial aggregation receptor 1 (PEAR1) and glycoprotein Ib platelet subunit alpha (GPIbα) [29, 30]. Additionally, we applied Ca^2+^ ionophore as a universal agonist for maximal (P)EV release [27].

In this study, we compared how PEVs induced through different platelet activation routes alter cancer hallmark functions. We applied PEV-educated 3D spheroids of two melanoma cell types with differential metastatic ability into multiple functional assays (proliferation, invasion, tube formation, cancer EV analysis) along with RNA sequencing to unravel the effects and signaling mechanisms relevant to melanoma progression and metastasis.

## Materials & methods

### Platelet isolation and activation

Standard leukocyte-reduced platelet concentrates, each derived from the buffy coats of four healthy donors sharing the same blood type (ABO RhD) were obtained from the Finnish Red Cross Blood Service (Helsinki, Finland) and handled anonymously, as accepted by the Finnish Supervisory Authority for Welfare and Health (Valvira, Helsinki, Finland). Platelet concentrates were prepared within 24 h of donation and the platelets were isolated by size-exclusion chromatography immediately upon arrival. Platelet concentration was measured with the Beckman Coulter T-540 (Beckman Coulter Inc., Brea, CA, USA) or Balio Diagnostics OX-360 hematology analyzers. Platelet concentration was adjusted to 2.5 × 10^8^ platelets/mL (physiological concentration in human blood) with Tyrode-HEPES buffer (137 mM NaCl, 0.3 mM NaH_2_PO_4_, 3.5 mM Hepes, 5.5 mM [D]-glucose, pH 7.35) supplemented with 2 mM CaCl_2_, 1 mM MgCl_2_, and 3 mM KCl. Platelets were activated with the addition of either (i) 0.5 µg/mL CRP (Triple Helical Peptides, UK), (ii) 250 µg/mL FFV (Sigma, St. Louis, MO, USA), (iii) 0.2 U/mL thrombin (Enzyme Research Laboratories Ltd., Swansea, UK) and 2 µg/mL collagen (HORM, Takeda Pharmaceuticals Co., Tokyo, Japan) co-stimulus (TC), or (iv) 10 µM Ca^2+^ ionophore (A23187, Sigma-Aldrich) (Ca^2+^) for 1 h in + 37 °C. After activation, platelets were centrifuged at 2500 x g for 15 min at room temperature (RT), after which the supernatant was transferred to a fresh tube and centrifuged again at 2500 x g for 15 min at RT [31]. Absence of residual platelets was verified with the hematology analyzer and the supernatants were directly used for PEV isolation.

### PEV isolation

The PEV containing supernatant was first concentrated through 10 kDa cut-off ultrafiltration devices (Amicon^®^ Ultra-15 Centrifugal Filter Unit, Millipore, Billerica, MA, USA) into 500 µL and then loaded onto 10 mL size-exclusion chromatography columns (Sepharose CL-2B, GE Healthcare). Four fractions of 500 µL (fractions 1–4) were collected after the void volume (2.85 mL) and concentrated into 100 µL with 10 kDa cut-off ultrafiltration devices (Amicon^®^ Ultra-4 Centrifugal Filter Unit, Millipore, Billerica, MA, USA). Particle counts were immediately measured with nanoparticle tracking analysis (NTA). Samples were stored in protein low binding tubes (Eppendorf SE, Germany) in several single use (10 µL) aliquots at -70 °C and used within 3 months of preparation.

### Nanoparticle tracking analysis (NTA)

Concentration and size of the EV samples were measured with ZetaView PMX-120 instrument (Particle Metrix, Germany) equipped with a 488 nm laser. Before measurement, the ZetaView was calibrated with polystyrene latex microbeads at 100 nm (Particle Metrix, Germany). Two cycles of measurements were performed for each sample, capturing at least 50 particles per frame. All settings were kept constant (focus: autofocus; camera sensitivity: 85; shutter: 100). Videos were analyzed by the ZetaView software version 8.05.16 SP3 to determine the concentration and size of measured particles. NTA post-acquisition settings were kept constant between samples (min size: 10; max size: 1000; min brightness: 30).

### Protein quantification

Protein content of the PEVs was measured with detergent compatible protein assay (Bio-Rad, USA) according to manufacturer’s instructions with bovine serum albumin as standard. First, PEVs were treated with 0.5% Triton X-100 (Sigma-Aldrich). Absorbances were measured at 750 nm with Multiscan GO spectrophotometer (Thermo Fisher Scientific, Waltham, MA, USA).

### Transmission electron microscopy (TEM)

TEM sample preparation and imaging were performed by the EV Core Facility, Institute for Molecular Medicine Finland (FIMM) Technology Centre, University of Helsinki. In brief, PEVs were loaded to carbon coated and glow discharged 200 mesh copper grids with pioloform support membrane. Then, PEVs were fixed with 2.0% paraformaldehyde in NaPO_4_ buffer, stained with 2% neutral uranyl acetate, embedded in uranyl acetate and methyl cellulose mixture (1.8/0.4%) and viewed with TEM Hitachi HT7800 operating at 100 kV. Images were taken with Gatan Rio9 bottom mounted CMOS-camera, model 1809 (Gatan Inc., USA) with 3072 × 3072 pixels image size and no binning.

### Cell lines

A2058 (ATTC^®^ CRL-11147™) and MV3 (RRID: CVCL_W280) cell lines were kindly donated by Docent. Sanna Pasonen-Seppänen (University of Eastern Finland) and cell line authentication was made by Genotyping Unit, FIMM Technology Centre, University of Helsinki. Cell lines characteristics are presented on Additional file: Supplementary Table 1. Human umbilical vein endothelial cells (HUVECs) were isolated as described previously [32] under the approval of the local ethics committee of the state of Upper Austria (ethics committee vote #200, 12/02/2005).

### Cell culture

Melanoma cell lines were cultured in Dulbecco’s modified eagle medium (Sigma-Aldrich, Media Kitchen, University of Helsinki) supplemented with 10% EV-depleted fetal bovine serum (FBS) (Gibco, Thermo Fisher Scientific, Waltham, MA, USA), 1% Penicillin-Streptomycin (5000 U/mL – Thermo Fisher Scientific), and incubated at 37 °C, 5% CO_2_, humidified atmosphere. HUVECs were cultured using EGM™-2 Endothelial Cell Growth Medium-2 BulletKit™ (Lonza, Walkersville, MD, USA) media and incubated at 37 °C, 5% CO_2_, humidified atmosphere. During the experiments, FBS component from EGM™-2 Endothelial Cell Growth Medium-2 BulletKit™ (Lonza, Walkersville, MD, USA) was replaced with 2% EV-depleted FBS (Gibco, Thermo Fisher Scientific, Waltham, MA, USA). EV-depleted FBS was prepared as previously described [33].

### Cell viability

MV3 and A2058 cells (50.000 cells/well) were seeded in 24-well plates. After 24 h, the culture medium or PEVs/cell in different ratios (1, 10, 100, 1000 PEVs/cell) were given at each corresponding well for 24 h. Later, the cells were harvested and counted by trypan blue dye exclusion. The data is shown by live cells counted/mL.

### Scratch assay

MV3 cells (200.000 cells/well) were seeded in 6-well plates. After 24 h, a line was made by scratching the bottom of the culture dish. The culture medium was removed and new culture medium or medium with PEVs (1000 PEVs/cell) was given to each corresponding well for 24 h. Wells were imaged at 0 h, 4 h and 24 h after the treatment using EVOS XL Core Cell Imaging System (Thermo Fisher Scientific). The area of the gap was measured immediately after the scratch (At = 0) and at subsequent time points (At = Δh). The percentage of closure was calculated using this formula: Gap Closure % = {(At = 0 – At = Δh)/At = 0} × 100, where At = 0 represents the initial gap area, and At = Δh is the gap area measured after h hours.

### Spheroid growth assay

MV3 (3500 cells/well) and A2058 (2500 cells/well) cells were seeded in ultra-low-attachment 96-well plates (Nunclon™ Sphera™ 96-Well, Nunclon Sphera-Treated, U-Shaped-Bottom Microplates, Thermo Fisher Scientific). After seeding, the plate was centrifuged at 300 x g for 5 min to collect all the cells to the bottom to allow formation of spheroids. Starting from day 1, spheroids were treated with PEVs (1000 PEVs/cell) for 10 days or left without treatment as a control. Treatments were repeated every 48 h. Spheroid growth was followed by microscopic images obtained (10x magnification) with EVOS XL Core Cell Imaging System (Thermo Fisher Scientific).

### Invasion assay

MV3 and A2058 cells (500 cells/well) were seeded in ultra-low-attachment 96-well plates (Nunclon™ Sphera™ 96-Well, Nunclon Sphera-Treated, U-Shaped-Bottom Microplates, Thermo Fisher Scientific) with a final volume of 200 µL/well. The ultra-low-attachment plates were centrifuged at 300 x g for 10 min and then incubated at 37 °C, 5% CO_2_, humidified atmosphere for 24 h to allow the spheroid formation. After 24 h, 100 µL of medium was removed from each well and spheroids were treated with PEVs. Finally, a layer of 0.7% GrowDex^®^-T anionic nanofibrillar cellulose (NFC) hydrogel (UPM Biomedicals, Finland) (100 µL) was added on top of the spheroids. Images of spheroids were acquired every 24 h for 3 days using EVOS XL Core Cell Imaging System (Thermo Fisher Scientific). Spheroid invasion was measured by calculating the difference in the area of the spheroids (day 3 - day 0).

### Tube formation assay

MV3 and A2058 cells (400.000 cells/well) were seeded in 6-well plates and treated for 16 h with PEVs (1000 PEVs/cell). The following day, a layer of Matrigel (BD Matrigel™, USA) was added to the 15-well angiogenesis chambers (µ-Slide Angiogenesis, ibidi, USA) and allowed to polymerize at 37 °C for 1 h. Later, treated cancer cells were harvested and mixed with HUVECs at a ratio of 2:1 (cancer cells: HUVECs) and seeded at a final concentration of 10.000 cells/well in the 15-well angiogenesis chambers coated with Matrigel. The wells were fixed with 4% paraformaldehyde for 15 min at the endpoint of the experiment and the wells were imaged at 5 h, 10 h, and 24 h. The total number and length of formed tubes in the endothelial network were quantified and analyzed by Fiji ImageJ software.

### Spheroid-derived EV isolation

3D culture was performed as described before [33]. Briefly, 1.5% (w/v) GrowDex^®^ NFC hydrogel (UPM Biomedicals, Helsinki, Finland) was diluted with experiment medium into working concentration 0.5% (w/v). After preparation of the NFC scaffolds, MV3 (35.000 cells/well) and A2058 (25.000 cells/well) cells were mixed with NFC hydrogel and 300 µL of the mixture was added per well into a 48-well plate. Spheroid morphology was regularly checked by imaging through EVOS XL Core Cell Imaging System (Thermo Fisher Scientific) (40x magnification). Every 48 h, the content of each well was resuspended, and centrifuged at 1000 x g (Fresco™ 21 Microcentrifuge, Thermo Scientific; 24 × 1.5/2.0mL rotor with ClickSeal™ Biocontainment Lid Fixed angle (45°), Thermo Scientific) for 10 min. The supernatant was collected for EV isolation and the pellet was mixed with fresh cell culture medium and seeded back into the 48-well plate. At day 10, the pellet was digested with 600 µg/mg (µg cellulase/mg cellulose) GrowDase^®^ (UPM Biomedicals, Helsinki, Finland) by incubating in 37 °C, 5% CO_2_ for 24 h. After digestion, the media was collected for EV isolation and cells were counted. EV isolation was performed by ultracentrifugation at 110.000 x g (Optima MAX XP Ultracentrifuge – TLA55 Fixed-Angle 45° Rotor, k-Factor 66) for 120 min at 4 °C. After centrifugation, the supernatant was discarded and the EV pellet was resuspended in 1 x dPBS (Sigma Aldrich, USA) and stored at -70 °C for experiments.

### Single particle interferometric reflectance imaging sensing (SP-IRIS)

The SP-IRIS ExoView^®^ R100 protocol was followed according to the manufacturer’s instructions (NanoView Biosciences, MA, USA), as described before [33]. Spheroid-derived EV samples were analyzed with the ExoView human tetraspanin kit (NanoView Biosciences, MA, USA). Additionally, the ExoView plasma tetraspanin kit was used to control the presence of residual PEVs in melanoma EV samples. Briefly, the samples were thawed and centrifuged at 2500 x g (Hettich MIKRO 200R centrifuge, fixed angle rotor [45°] 24-places), 15 min, at RT. Next, the samples were diluted in solution B based on NTA particle concentrations (3.5 × 10^8^ particles for PEV samples and 3.5–3.8 × 10^8^ particles for spheroid-derived EV samples) and 35 µL of sample was added directly on the chip and incubated at RT for 16 h. The chips were then incubated with the labelling antibodies anti-cluster of differentiation (CD) 81 Alexa-555, anti-CD63 Alexa-488, and anti-CD9 Alexa-647. After incubation, chips were washed, dried, and scanned for interferometric reflectance imaging and fluorescent detection with the ExoView R100 reader. The data were analyzed using ExoViewer 2.5.0 with sizing thresholds set from 50 to 200 nm.

### RNA isolation

MV3 (35.000 cells/well) and A2058 (25.000 cells/well) spheroids were grown in 0.5% (w/v) GrowDex^®^ NFC hydrogel (UPM Biomedicals, Helsinki, Finland) in 48-well plates for 5 days. PEV treatment (1000 PEVs/cell) was given every 48 h. At day 5, the spheroid pellet was digested with 600 µg/mg (µg enzyme/mg cellulose) GrowDase^®^ cellulase enzyme mix (UPM Biomedicals, Helsinki) by incubating at 37 °C in humidified 5% CO_2_ atmosphere for 8 h. The last PEV treatment was given for 6 h or 24 h with fresh medium. After each time point, spheroids were pelleted down by centrifugation at 1000 x g for 10 min at 4 °C and frozen inside a Trizol reagent at -70 °C for RNA isolation. The quantity and quality of RNA in the samples were evaluated with the Nanodrop 2000c spectrophotometer (Thermo Fisher Scientific), and Agilent RNA 5400 Nano Chips (Agilent Technologies).

### RNA-sequencing (RNA-seq)

Briefly, mRNA was purified from total RNA using poly-T oligo-attached magnetic beads. After fragmentation, the first strand complementary DNA (cDNA) was synthesized using random hexamer primers, followed by the second strand cDNA synthesis using deoxyuridine triphosphate and deoxythymidine triphosphate. After cluster generation, the library preparations were sequenced on an Illumina Novaseq platform and 150 base paired-end reads were generated (Novogene, Cambridge, UK). Differentially expressed genes between each treatment and the control samples were identified using DESeq2 R package (1.20.0) (absolute log2 fold change > 1 and adjusted p value < 0.05). Genes with an adjusted p-value < = 0.05 (Benjamini and Hochberg) were assigned as differentially expressed.

### Enrichment analysis of differentially expressed genes (DEGs)

Gene Ontology (GO) (https://geneontology.org/) and Kyoto Encyclopedia of Genes and Genomes (KEGG) (http://www.genome.jp/kegg/) enrichment analyses of differentially expressed genes were analyzed by the cluster Profiler R package, in which gene length bias was corrected. GO and KEGG pathways were considered significantly enriched by differentially expressed genes when adjusted p value was < 0.05.

### Quantitative reverse transcription PCR (RT-qPCR)

Total RNA was converted into cDNA using the iScript™ cDNA Synthesis Kit (Bio-Rad, USA). Real-time PCR was carried out in triplicates using the SsoAdvanced Universal SYBR Green Supermix (Bio-Rad, USA) and CFX Opus 96 Real-Time PCR System (Bio-Rad, USA). RT-qPCR primers were designed through Primer BLAST platform (Additional file: Supplementary Table 2). Fold change of each selected gene was normalized to GAPDH. BioRad CFX Manager 3.1 was used for data analysis.

### Data analysis

All data are expressed as the means ± SD from at least three independent experiments. The Shapiro-Wilk test was used to test the datasets for normal distribution. One-way analysis of variance (ANOVA), Kruskal-Wallis test, two-way ANOVA, or Welch’s t test was used for the analyses (GraphPad Prism 9.0). * = *p* < 0.05, ** = *p* < 0.01, *** = *p* < 0.001, **** = *p* < 0.0001 was considered to be statistically significant when compared with the control groups. All microscopy image analyses were performed by Fiji ImageJ software.

## Results

### Platelet activation pathway determines the potency of PEVs to promote the growth of melanoma spheroids

To generate GPVI PEVs, GPVI was activated with a receptor-specific peptide, CRP. As a conventional, well-established PEV-inducing signal, thrombin and collagen were used together to co-activate all thrombin and collagen receptors producing TC PEVs. Ca^2+^ ionophore was applied as a potent, but non-receptor-specific, vesiculation inducer generating Ca^2+^ PEVs [27]. Additionally, we employed fucoidan from *Fucus vesiculosus* to activate the CLEC-2 receptor (along with PEAR1 and GPIbα receptors producing FFV PEVs [30]). As FFV was a previously undescribed PEV-inducing agonist, we characterized the resulting PEVs according to the Minimal Information for Studies of Extracellular Vesicles (MISEV) 2023 guidelines [1]. The FFV-induced PEVs presented similar size, morphology, and expression of the three common EV tetraspanin markers (CD9, CD63, CD81) as the previously characterized CRP, TC and Ca^2+^ PEVs [9, 27] (Additional file: Fig S1). However, just like CLEC-2 -specific snake venom rhodocytin [9], FFV was a weak inducer of PEV formation in terms of number of particles, but the protein content of FFV PEVs was high and comparable to that of TC and Ca^2+^ PEVs (Additional file: Fig S1B).

Next, we analyzed the dosage of PEVs, given that cells respond differently to low and high EV doses [34]. A ratio of 1000 PEVs/melanoma cell was sufficient to generate a statistically significant increase in proliferation in conventional 2D cultures (Additional file: Fig S2A). Since only Ca^2+^ PEVs enhanced proliferation, we also tested the PEVs in a 2D scratch assay, where none of the PEVs had an effect in wound closure (Additional file: Fig S2B). Based on these results, 1000 PEVs/melanoma cell ratio was maintained in the subsequent 3D functional assays.

**Fig. 1:**
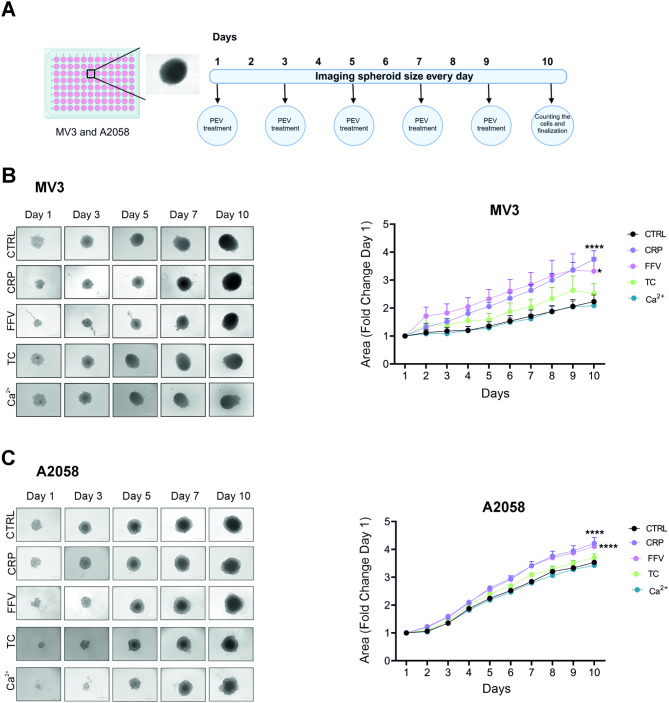
MV3 and A2058 spheroid growth upon differential platelet-derived extracellular vesicle (PEV) treatments. **A** Spheroids from MV3 and A2058 were grown in ultra-low attachment plates and treatment with or without PEVs (1000 PEVs/cell) was repeated every 48 h for 10 days. **B-C** The spheroids were imaged by an inverted microscope every day to measure the growth, and fold change was calculated compared to day 1 (*n* = 3; biological replicates representing 12 donors, Statistics: two-way ANOVA followed by Dunnett’s multiple comparisons test, * = *p* < 0.05, ** = *p* < 0.01, *** = *p* < 0.001, **** = *p* < 0.0001)

Since the in vivo-like cell environment of tumors is better mimicked by 3D spheroids than 2D cell cultures, we applied our recent method of 3D cancer spheroid culture which implements a novel NFC matrix-based approach [33]. To evaluate the effect of the different PEVs on melanoma spheroid growth, the PEVs were added to the spheroids every 48 h for 10 days (Fig. 1A). Image analysis of the MV3 and A2058 spheroids showed that both CRP PEVs and FFV PEVs markedly boosted spheroid growth, followed by TC PEVs. In contrast, there was no effect from Ca^2+^ PEVs when compared to the untreated spheroids (Fig. 1B-C). Both CRP and FFV PEV treatments enhanced the spheroid growth of MV3 and A2058 spheroids statistically significantly when compared to the untreated spheroids.

### CRP and FFV PEVs enhance invasiveness of the melanoma spheroids

**Fig. 2:**
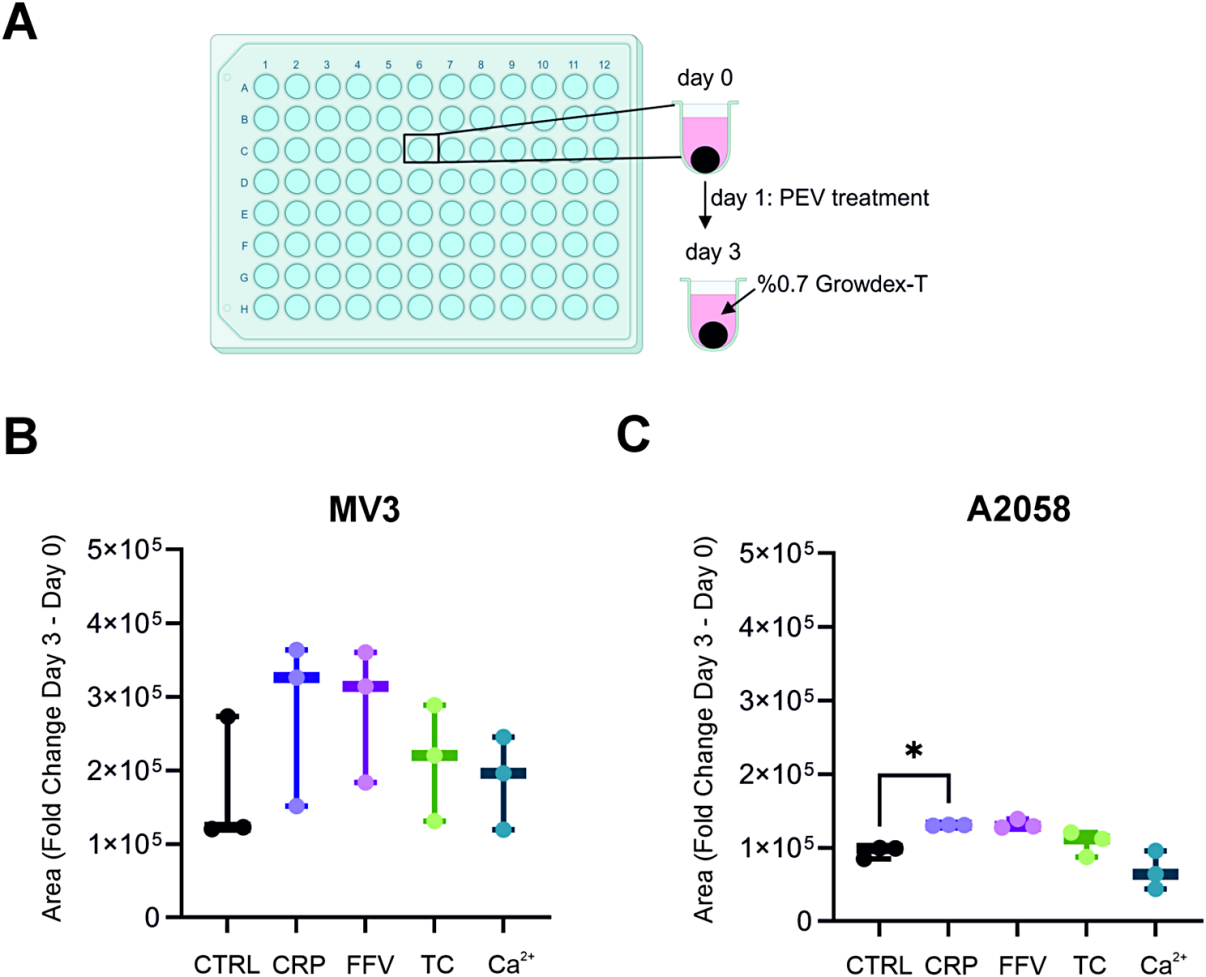
Invasiveness of MV3 and A2058 spheroids upon differential platelet-derived extracellular vesicle (PEV) treatments. **A** MV3 and A2058 spheroids were grown in ultra-low attachment plates, and PEV treatment (1000 PEVs/cell) was given on day 1. A layer of 0.7% NFC hydrogel was added on top of the spheroids, and the spheroids were allowed to invade until day 3. **B-C** The differences in the spheroid surface area were calculated between day 3 and day 0 for the MV3 and A2058 spheroids (*n* = 3; biological replicates representing 12 donors, Statistics: Kruskal-Wallis test, * = *p* < 0.05)

Next, we evaluated the effects of differentially induced PEVs on the invasiveness of MV3 and A2058 spheroids. For this purpose, single spheroids were formed in ultra-low attachment 96-well plates with 0.7% anionic NFC hydrogel as a growth support. A single PEV treatment was administered before the analysis on day 3 (Fig. 2A). Both CRP and FFV PEVs seemed to increase the invasive capacity of MV3 and A2058 spheroids in a similar manner (Fig. 2B-C), but statistical significance was only reached with CRP PEVs in A2058 spheroids. Again Ca^2+^ and TC PEVs had negligible effects on invasiveness and were comparable to untreated controls.

### Endothelial tube formation is minimally altered by differentially induced PEVs in melanoma cells

**Fig. 3:**
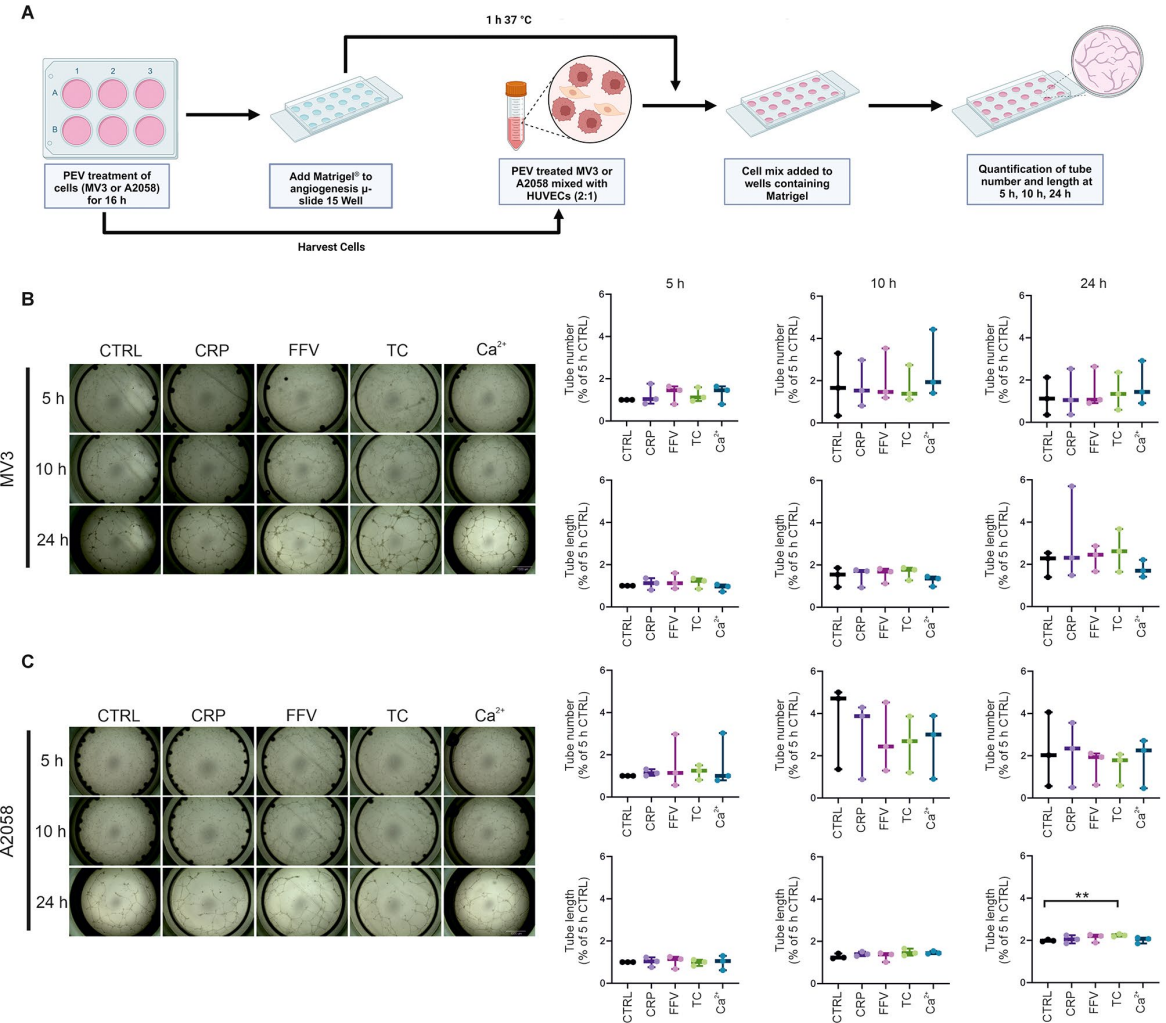
Pro-angiogenic potential of MV3 and A2058 cells upon differential platelet-derived extracellular vesicle (PEV) treatments. **A** MV3 or A2058 cells were seeded onto 6-well plates and treated with PEVs for 16 h. Subsequently, the PEV-pretreated cancer cells were mixed with HUVECs in 2:1 ratio and added to angiogenesis chambers containing Matrigel. **B**-**C** Imaging to quantify the number and average length of the tubes in the endothelial network was performed at 5 h, 10 h and 24 h. (*n* = 3; biological replicates representing 12 donors, Statistics: Welch’s test, * = *p* < 0.05, ** = *p* < 0.01)

As the third cancer hallmark function, we analyzed the pro-angiogenic potential of the PEV-pretreated melanoma cells mixed with HUVECs in a tube formation assay. The number and length of the newly formed tubules in the endothelial network was analyzed at 5 h, 10 h, and 24 h (Fig. 3A). No statistically significant differences were found in the number or length of the formed vessel tubes with any of the PEV-pretreated MV3 cells when they were co-cultured with the HUVECs (Fig. 3B). Only the TC PEV-pretreatment of A2058 cells significantly increased the tube length, but not their number, at 24 h (Fig. 3C).

In summary, the results of our 3D functional assays, proliferation and invasion, showed that PEVs were tunable by platelet activation, and altered the cancer hallmark functions differently depending on their induction route and the type of the cancer cells.

### PEVs alter the vesiculation of melanoma spheroids

**Fig. 4:**
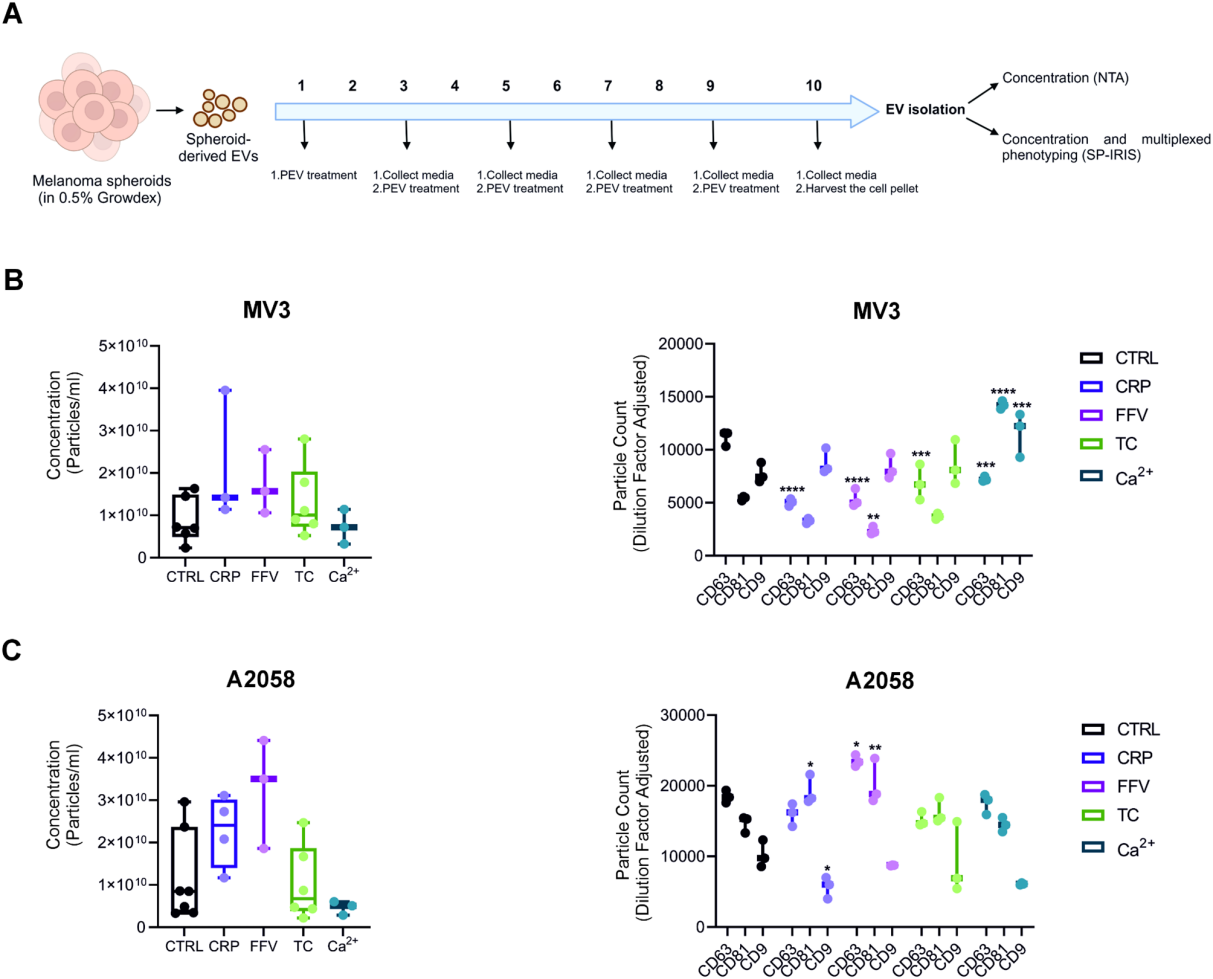
Platelet-derived extracellular vesicles (PEVs) differentially alter the number and tetraspanin profiles of EVs from melanoma spheroids. **A** Experimental workflow for melanoma EV isolation and characterization. **B**-**C** Particle concentration (p/mL) was measured by NTA and SP-IRIS was used for concentration and tetraspanin (CD63, CD81, CD9) profiling for MV3 and A2058 spheroid-derived EVs. (*n* = 6; biological replicates representing 24 donors for CTRL and the TC PEV-treated spheroids, *n* = 3; biological replicates representing 12 donors for the CRP, FFV and Ca^2+^ PEV-treated spheroids, Statistics: one-way ANOVA, Kruskal- Wallis test, two-way ANOVA followed by Dunnett’s multiple comparisons test, * = *p* < 0.05, ** = *p* < 0.01, *** = *p* < 0.001, **** = *p* < 0.0001)

Given that cancer EVs play a crucial role in tumor microenvironment and pre-metastatic niche formation [2, 35], we sought to investigate whether PEVs would affect the EV secretion of melanoma cells. To create a more relevant 3D environment, we grew the spheroids in NFC hydrogel according to our novel method [33]. The absence of residual PEVs in the cancer EV samples was verified by the absence of the platelet marker CD41a in the melanoma spheroid EV samples by SP-IRIS (Additional file: Fig S3). After PEV treatment of the spheroids of both melanoma cell types, we analyzed their EV secretomes by changes in the particle number (NTA) and tetraspanin signature (SP-IRIS) (Fig. 4A). No statistically significant differences in particle numbers were observed with any of the PEV treatments but the most notable effects were again observed with CRP and FFV PEVs, while TC and Ca^2+^ PEVs had no effect in MV3 and A2058 spheroids (Fig. 4B-C).

In contrast, the profiles of the melanoma-derived EVs were altered by all PEV types, as revealed through SP-IRIS analysis of the EV tetraspanin signatures. All PEV types significantly decreased CD63-expressing EVs from the MV3 spheroids. Further, Ca^2+^ PEVs significantly increased the number of CD81- and CD9-containing EVs from MV3 spheroids, whereas FFV PEVs decreased the CD81-containing EVs from MV3 spheroids compared to EVs from untreated MV3 spheroids (Fig. 4B). In the A2058 spheroids, CRP PEVs significantly increased CD81- and decreased CD9-containing melanoma EV formation, whereas FFV PEVs increased the formation of CD63- and CD81-containing melanoma EVs (Fig. 4C).

In summary, all PEVs were able to modulate the functionality of both melanoma spheroid types based on the molecular profiles of the secreted cancer EVs, but not by EV numbers.

### PEVs differently modulate multiple signaling pathways in MV3 spheroids

**Fig. 5:**
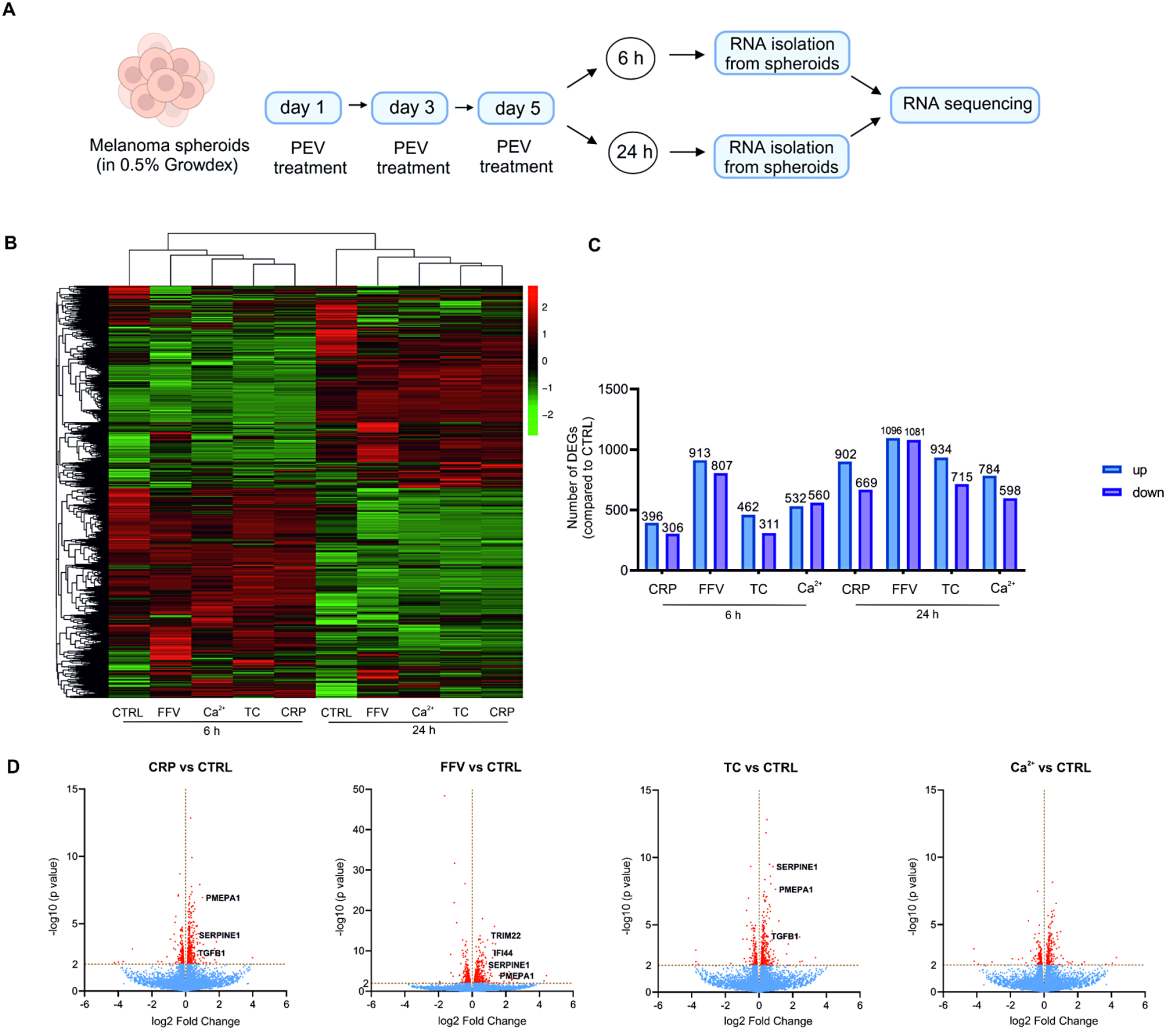
RNA-sequencing (RNA-seq) of the MV3 spheroids treated with different platelet-derived extracellular vesicles (PEVs). **A** Melanoma spheroids were grown inside 0.5% NFC hydrogel for 5 days as previously described [33]. PEV treatment was repeated three times every 48 h. On day 5, the PEV treatment lasted either 6 h or 24 h before RNA isolation to observe temporal changes in the transcriptome. **B** Hierarchical clustering of gene expression values for 6 h and 24 h treatments. Red color indicates genes with high expression levels, and green color indicates genes with low expression levels. **C** Number of differentially expressed genes (up- and downregulated) for the CRP, FFV, TC and Ca^2+^ PEV treatments compared to untreated spheroids at 6 h and 24 h. **D** Volcano plots showing the –log10 (p value) vs. log2 fold change differences in the CRP, FFV, TC and Ca^2+^ PEV-treated melanoma spheroids compared to untreated spheroids at 24 h. Red dots indicate the significant genes while blue dots indicate non-significant genes. The genes used in RT-qPCR for validation are highlighted in bold (*n* = 4; biological replicates representing 16 donors, Statistics: Benjamini and Hochberg, DESeq2 pvalue < = 0.05, |log2FoldChange|>=0.0)

Since PEVs seemed to differ in their efficacy in altering the cancer functional properties of the MV3 and A2058 spheroids, we aimed to identify which molecular pathways were modulated. We first employed RNA-seq on the more metastatic MV3 melanoma spheroids treated with CRP, FFV, TC, and Ca^2+^ PEVs on days 1, 3 and 5. To detect possible temporal changes in gene expression, we also isolated RNA either 6 h or 24 h after the last PEV treatment on day 5 (Fig. 5A). Heat map analysis of the differentially expressed genes (DEGs) revealed that indeed the time point difference (6 h vs. 24 h) was a main factor for clustering together genes with similar expression patterns (Fig. 5B). The number of DEGs was higher at the 24 h time point compared to the 6 h time point for all PEV treatments (Fig. 5C). The majority of the genes were commonly expressed between the PEV-treated and untreated spheroids (Additional file: Fig S4), but we also observed significant PEV type -dependent alterations when comparing the number of DEGs in the PEV-treated MV3 spheroids to untreated spheroids (Fig. 5C). The FFV PEV treatment generated the highest number of DEGs and the most significant ones, while the Ca^2+^ PEVs induced the fewest and least significant DEGs compared to untreated MV3 spheroids (Fig. 5D).

Two commonly expressed genes, serine protease inhibitor family E member 1 (SERPINE1) and prostate transmembrane protein, androgen induced 1 (PMEPA1), along with other highlighted genes (IFI44, TRIM22 and TGFB1) from the volcano plots at 24 h (Fig. 5D) as well as SMAD6 and NOX4 from the 6 h time point, were next validated by RT-qPCR (Additional file: Fig S5). The fold changes of SERPINE1 and PMEPA1 from the RNA-seq and RT-qPCR were consistent in terms of upregulation or downregulation for all the PEV treatments at both time points. For example, RNA-seq showed that SERPINE1 was upregulated 1.93-fold and 1.78-fold with the TC PEV treatment at 6 h and 24 h, respectively, and the RT-qPCR of SERPINE1 showed 1.97- and 1.88- fold increase at 6 h and 24 h, respectively. RT-qPCR also confirmed the total absence of the SERPINE1 in the Ca^2+^ PEV-treated MV3 spheroids and the temporal differences in the PMEPA1 gene expression (at 6 h, the fold changes for the FFV PEV-treated MV3 spheroids were 5.89 in RNA-seq and 9.36 in RT-qPCR, respectively. At 24 h, PMEPA1 gene expression was no longer detectable for the same PEV treatment both by RNA-seq and RT-qPCR). The fold changes of all the genes from the RNA-seq and RT-qPCR are presented in Additional file: Supplementary Table 3. Given that the most significant DEGs were observed at 24 h in the CRP and FFV PEV-treated spheroids, we focused our pathway enrichment analyses on these PEV-treatments at this time point.

**Fig. 6:**
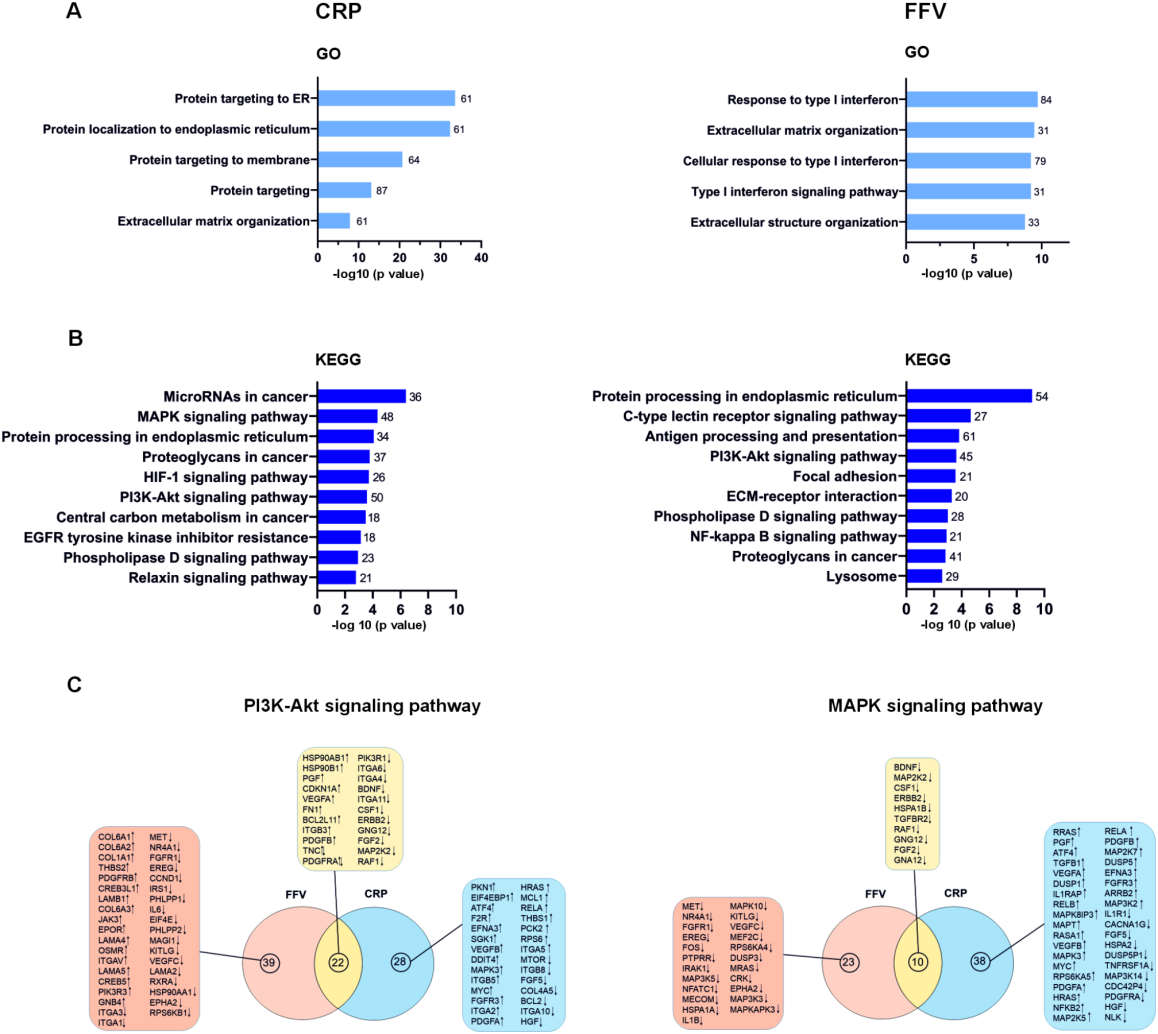
Gene Ontology (GO) and Kyoto Encyclopedia of Genes and Genomes (KEGG) pathway analyses of the CRP PEV and FFV PEV-treated MV3 spheroids for 24 h. **A**-**B** On the x-axis, -log10 (p value) and on the y-axis, GO or KEGG terms are given. The number of DEGs that are significantly different (compared to untreated spheroids) is indicated at the end of the GO or KEGG term columns. The significant DEGs indicated by the CRP PEVs were enriched in protein targeting/processing in ER, ECM organization, microRNAs in cancer, MAPK signaling pathway, proteoglycans in cancer, HIF-1 and PI3K-Akt signaling pathways and central carbon metabolism in cancer. The significant DEGs indicated by the FFV PEVs were enriched in type-I IFN related pathways, ECM organization, and protein processing in ER, C-type lectin receptor signaling pathway, antigen processing and presentation, PI3K-Akt signaling pathway, focal adhesion and ECM-receptor interaction. **C** Common and unique DEGs in the PI3K-Akt and MAPK signaling pathway in the CRP and FFV PEV-treated spheroids. Upregulated and downregulated genes are indicated by the direction of arrow and arranged from the most to the least significant. (*n* = 4; biological replicates representing 16 donors, Statistics: Benjamini and Hochberg, adjusted p-value < = 0.05)

GO enrichment analysis was applied to reveal the biological significance of the DEGs induced by each PEV treatment in the MV3 spheroids. CRP PEVs most significantly activated signaling pathways of protein targeting to the endoplasmic reticulum (ER) and membrane, and organization of the extracellular matrix (ECM) components like matrix metalloproteinase (MMP) 14, MMP16, collagen type V alpha 1 chain, versican (Fig. 6A). The effects on protein targeting to the ER and membrane were already evident at the 6 h time point (Additional file: Fig S6A). ECM reorganization was also significantly activated in the FFV PEV-treated MV3 spheroids, but not at 6 h when interestingly, the most significant GO functions were cell motility, migration, angiogenesis and wound healing (Additional file: Fig S6A). Completely different from the CRP or other PEV treatments, FFV PEVs uniquely activated type I interferon (IFN-I) related signaling pathways and responses to IFN-I (Fig. 6A).

KEGG pathway enrichment analysis was also performed with the significant DEGs from each PEV treatment, and comparisons were made against the untreated spheroids. The most significant differences in the enriched pathways were again induced by CRP and FFV PEVs. The enriched pathways indicated by the significant DEGs in the CRP PEV-treated spheroids were microRNAs in cancer, mitogen-activated protein kinase (MAPK) signaling pathway, protein processing in the ER, proteoglycans in cancer, HIF-1, and PI3K-Akt signaling pathways, central carbon metabolism in cancer, EGFR tyrosine kinase inhibitor resistance, phospholipase D and relaxin signaling pathways (Fig. 6B). Interestingly, oxidative phosphorylation and transforming growth factor beta (TGF-β) signaling pathways were only enriched at 6 h by CRP PEVs (Additional file: Fig S6B). The enriched pathways indicated by the significant DEGs in the FFV PEV-treated MV3 spheroids were protein processing in the ER, C-type lectin receptor signaling pathway, antigen processing and presentation, phosphotylinosital 3 kinase/ protein kinase B (PI3K-Akt) signaling pathway, focal adhesion, ECM-receptor interaction, phospholipase D and NF-kappa B signaling pathways, proteoglycans in cancer and lysosome (Fig. 6B). The C-type lectin receptor signaling pathway and antigen presentation were only induced by the FFV PEV treatment, supporting our strategy to engage CLEC-2 receptor. PI3K-Akt signaling pathway, focal adhesion, ECM-receptor interaction and proteoglycans in cancer were common pathways at 6 h and 24 h in the FFV PEV-treated MV3 spheroids (Additional file: Fig S6, Fig. 6B). The other enriched pathways indicated by the significant DEGs in the FFV PEV-treated MV3 spheroids at 6 h were Hippo signaling pathway, microRNAs in cancer, hepatocellular carcinoma, Wnt signaling pathway, gastric cancer and basal cell carcinoma. Common pathways induced by both the FFV and CRP PEVs were the PI3K-Akt signaling pathway, protein processing in the ER and phospholipase D signaling pathway (Fig. 6B). Unique pathways induced by the CRP PEV treatment included the HIF-1 signaling pathway, central carbon metabolism in cancer, EGFR tyrosine kinase inhibitor resistance and relaxin signaling pathway. Unique pathways induced by the FFV PEV treatment included C-type lectin receptor signaling pathway, antigen processing and presentation, focal adhesion, ECM-receptor interaction, NF-kappa B signaling pathway and lysosome. Interestingly, downregulated DEGs induced by the FFV PEVs were significantly enriched in the MAPK signaling pathway (Fig. 6C).

### CRP and FFV PEVs alter the MAPK signaling and energetic metabolism pathways in A2058 spheroids

Based on the GO and KEGG pathway analyses of the RNA-seq, CRP and FFV PEVs were identified as the most active modulators of the MV3 transcriptome. To elaborate especially the immunomodulatory impact of PEVs on melanoma, we performed another RNA-seq with the CRP and FFV PEV-treated A2058 spheroids at 6 h and 24 h (Fig. 5A) and compared the results with untreated spheroids. Similarly to the MV3 results, the primary clustering factor of genes was the time point (Additional file: Fig S7A). However, in contrast to the MV3 spheroids, the most significant changes were observed at 6 h (with both the CRP and FFV PEVs), instead of 24 h as with the MV3. Overall, both the CRP and FFV PEV-treated A2058 spheroids exhibited fewer significant DEGs when compared to the results from the MV3 spheroids (Additional file: Fig S7B-C).

GO analysis of the significant DEGs in the A2058 spheroids treated with the CRP and FFV PEVs for 6 h revealed that both PEVs activated mitochondrial metabolism related pathways. Additionally, FFV PEVs affected ECM and MAPK signaling pathways (Additional file: Fig S8A). KEGG pathway analysis showed that oxidative phosphorylation and chemical carcinogenesis (reactive oxygen species) were significantly downregulated by CRP and FFV PEVs at 6 h (Additional file: Fig S8B). In line with the impacted pathways in the MV3 spheroids, the PI3K-Akt signaling pathway was significantly upregulated by the FFV PEV treatment at 6 h. However, in contrast to the MV3 spheroids, the effect was gone at 24 h indicating temporal differences between the cell types. Interestingly, further temporal changes in the DEGs were observed, when we compared the same PEV treatments at 6 h and 24 h. In the CRP PEV-treated A2058 spheroids, pathways of cell cycle, microRNAs in cancer, MAPK signaling, proteoglycans in cancer and platelet activation were significantly upregulated at 6 h compared to 24 h. In the FFV PEV-treated A2058 spheroids, cell cycle, platelet activation, regulation of actin cytoskeleton, focal adhesion and PI3K-Akt signaling pathways were significantly upregulated at 6 h compared to 24 h (Additional file: Fig S8C). This shows that the PEVs’ impact on the melanoma transcriptome depends on multiple factors: time, cell type and the platelet activation pathway of PEV induction.

## Discussion

**Fig. 7:**
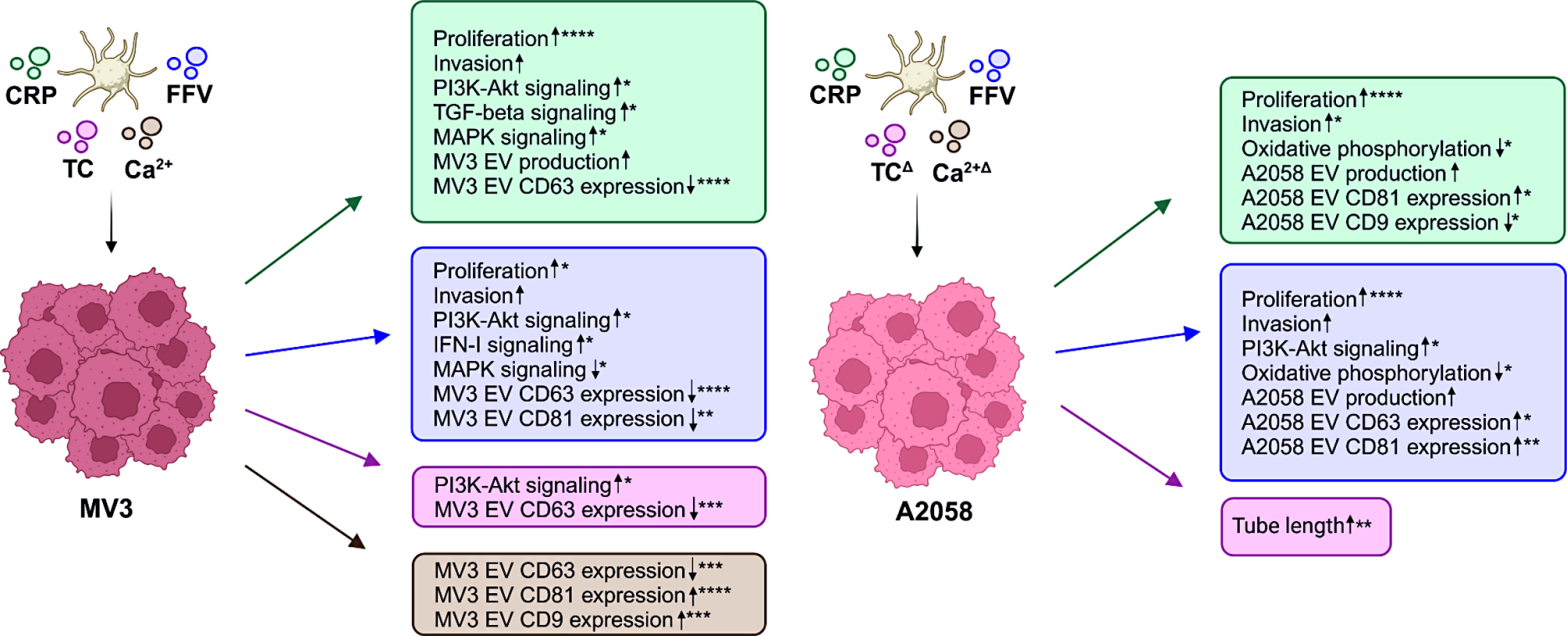
Schematic summary of the effects of PEVs on the MV3 and A2058 spheroids. CRP and FFV PEVs promote melanoma cancer hallmark properties by increasing proliferation, invasion, EV production, and they modulate the PI3K-Akt and MAPK signaling pathways. Specifically, CRP and FFV PEVs upregulate the TGF-β and IFN-I signaling pathways in the MV3 spheroids, respectively. Each PEV type is indicated by a color (CRP: green, FFV: purple, TC: pink, Ca^2+^: brown). * = *p* < 0.05, ** = *p* < 0.01, *** = *p* < 0.001, **** = *p* < 0.0001, Δ: the PEVs that were used only in functional assays in the A2058 spheroids

Cancer patients often suffer from thrombocytosis and are at a higher risk developing venous thromboembolism [36]. Increased platelet activity has also been associated with inflammatory responses and drug resistance in cancer patients [37, 38], and it is considered that platelets become more activated and release a greater amount of soluble factors, including EVs [39]. In this study, the impact of PEVs induced through different platelet signaling receptors was investigated using melanoma as a cancer model, since melanoma, the most aggressive form of skin cancer, can metastasize to distant organs via the bloodstream. To the best of our knowledge, previous research studies have explored PEVs generated from platelets using only a single agonist, commonly thrombin [11–13, 40–42]. Here, we employed four different platelet agonists to generate (i) CRP PEVs (GPVI receptor induced), (ii) FFV PEVs (CLEC-2 receptor induced), (iii) TC PEVs (thrombin- and collagen- pan activation), and (iv) Ca^2+^ PEVs (universal signal for receptor unspecific EV release). We were particularly interested in the two immunomodulatory receptors, GPVI and CLEC-2, given their recent identification as promising therapeutic targets in melanoma [25, 43]. GPVI and CLEC-2 have been shown to interact with e.g., galectin-3 and podoplanin, respectively, both of which are upregulated in melanoma [43, 44]. We used 3D spheroids of two melanoma cell types educated with the respective PEVs and assayed multiple cancer hallmark relevant cell functions (proliferation, invasion, pro-angiogenic potential, and melanoma EV production), including high-throughput RNA-seq, to unravel the individual PEV-affected molecular mechanisms. MV3 is a highly invasive and metastatic BRAF (B-Raf serine-threonine kinase)/RAS wild type cell line [45], and A2058 is aggressive but less metastatic BRAF^V600E^ mutated cell line (RRID: CVCL_1059) [46]. In both cell types, cancer hallmark functions and transcriptomes were differently altered depending on the PEV induction route. Regarding the transcriptomes, both temporal and cell type -specific changes were observed. A summary of all functional and transcriptomic alterations, along with their statistical differences based on the PEV type, is provided in Fig. 7.

Out of the PEV types, CRP and FFV PEVs were systematically the most active modulators of both melanoma cell types, although statistical significance was not reached in all the functional assays. CRP and FFV PEVs significantly enhanced proliferation in both melanoma cell types, while CRP PEVs significantly increased invasion in A2058 spheroids. As a common signaling pathway previously shown to be impacted by platelets in melanoma and breast cancer [47], FFV PEVs increased the PI3K-Akt signaling activity in the MV3 and A2058 spheroids. PI3K-Akt, together with the MAPK pathway, has been shown to be altered in melanoma, and these signaling routes also contribute to high mutation frequency in melanoma [48, 49]. Interestingly, in melanoma, the PI3K-Akt and MAPK pathways may function in opposing ways, such that one is suppressed while the other one is activated [50]. We found that in the MV3 spheroids, the MAPK pathway was upregulated by CRP PEVs, whereas FFV PEVs significantly downregulated it. Since both types of PEVs promoted proliferation and invasion, similarly to what has been reported for platelets [5], these findings underscore the complexity of the PEV-affected signaling pathways. Upregulated MAPK signaling in different cancer cell lines by platelets has been previously reported. For example, thrombin-induced PEVs promoted migration and invasiveness of highly metastatic MDA-MB-231 breast cancer cells by stimulating the phosphorylation of p38 MAPK and the myosin light chain 2 [12, 13]. Another study showed that thrombin-induced PEVs enhanced colorectal cancer cell invasiveness through the p38 MAPK pathway [11].

Another cancer mechanism-related observation, the downregulation of oxidative phosphorylation in the A2058 spheroids by CRP and FFV PEVs at 6 h (and in the MV3 spheroids by CRP PEVs at 6 h) might be indicative of the Warburg effect, wherein cancer cells preferentially shift from oxidative phosphorylation to glycolysis to optimize nutrient acquisition [51]. This metabolic adaptation is generally linked to increased resistance to apoptosis and tumor aggressiveness [52] and it might also be driven by the upregulation of the MAPK signaling pathway in the BRAF-mutant cells [53], such as the A2058 cell line. In this context, targeting the MAPK pathway could be especially critical for patients with BRAF mutations who develop resistance to BRAF/ mitogen-activated protein kinase kinase (MEK) inhibitors and immunotherapies [53]. Finally, both CRP and FFV PEVs significantly upregulated gene expression of ECM components (e.g. MMP14, MMP16, collagen type V alpha 1 chain, versican) as well as the genes SERPINE1 and PMEPA1, which are associated with epithelial-to-mesenchymal transition in various cancer types [54–56]. SERPINE1 expression has been reported to discriminate site-specific metastasis in human melanoma [57]. SERPINE1 codes plasminogen activator inhibitor 1 (PAI-1) protein expression. PAI-1 is widely expressed in skin cancers, and it may act on multiple levels as an anti-fibrinolytic and a modulator of ECM and MMPs underlying tumor microenvironment remodeling and melanoma invasion [58]. Recently, PAI-1 has also been identified as a regulator of programmed death-ligand 1 (PD-L1) expression, and its adjuvant inhibition may improve anti-PD-L1 therapy in melanoma [59, 60]. PMEPA1 protein regulates TGF-β [56], and although its role in melanoma is complex, it is generally considered to promote tumor progression [61].

In contrast to the effects induced by CRP and FFV PEVs, only minor statistically significant changes were observed in the transcriptomes and functional assays by TC and Ca²⁺ PEVs. TC PEVs mediated induction of genes in the PI3K-Akt pathway in the MV3 spheroids similar to CRP and FFV PEVs, whereas Ca^2+^ PEVs only led to alterations in the tetraspanin profiles of cancer EVs from the MV3 spheroids. The only statistically significant alteration in functionality by TC PEVs was the increase in the length of the tubes in the endothelial network when the PEV pre-treated A2058 cells were co-cultured with HUVECs. Although RNA-seq data showed that CRP PEVs and FFV PEVs activated angiogenesis-related genes in both cell types, they did not affect endothelial tube formation. While there are studies demonstrating a pro-angiogenic role of PEVs on HUVECs [62–64], as far as we are aware, none have investigated the pro-angiogenic potential of the PEV-pretreated melanoma cells co-cultured with HUVECs. In the study of Martini et al. [65], only inhibitory effects of both direct platelet contact and their secretomes (likely including PEVs) were found on vascular mimicry of melanoma cells, consistent with the lack of a pro-angiogenic effect observed in this study. Further research, preferably with animal models and multiple experimental settings (e.g. dosage and time), will be needed to explore the true potential of the PEV types on different types of cancer cells, to overcome the limitations of this study.

Transcriptomics uncovered significant temporal changes not only when comparing the PEV-treated vs. untreated spheroids at 6 h and 24 h, but also when comparing the different time points within the same treatment, showing that the impact of PEVs on cancer gene expressions is dynamic. It is currently not yet methodologically possible to determine whether temporal transcriptomic changes depend on differences in PEV uptake, cargo release and subcellular localization [66], or the transcriptomic re-programming potential of the cell. We think that the latter is more likely as we found both genes that showed temporal alterations including up- and downregulation and those that do not. This result highlights the relevance of monitoring transient signaling at multiple time points for comprehensive understanding of the effects of (P)EVs or other interventions, since most studies have employed (PEV) treatments at a single time point, typically 24 h [12, 13, 42].

PEVs also influenced the melanoma spheroids’ own EV production by modifying their tetraspanin (CD63, CD9, and CD81) profiles. Cancer cell-derived EVs are thought to affect multiple cell types (e.g. fibroblasts, macrophages, natural killer cells) at the primary tumor site and in distant organs, where they may facilitate pre-metastatic niche formation according to the “seed and soil” theory [2]. The fact that PEVs may alter cancer EV production may indicate their mechanistic contribution to metastatic colonization. Tetraspanins, such as those considered to be EV markers, have been reported to promote cancer progression and metastasis [1, 67, 68]. All the PEV treatments decreased CD63-containing cancer EVs compared to the EVs from untreated MV3 spheroids. This finding is interesting since decreased expression of CD63 (e.g. expelled into EVs) is an indication of increased motility and matrix degrading capabilities in melanoma [69]. Furthermore, PEV-dependent CD9- and CD81-containing EV modulation was found in the MV3 and A2058 spheroids. Overall, we observed tuning of the EV production and their tetraspanin signatures by different PEVs, possibly granting melanoma cells novel functional EV-mediated properties.

Use of two cancer-relevant receptors (GPVI and CLEC-2) to induce PEVs revealed major changes also in the PEV-dependent immunomodulatory responses in the melanoma cells. Upregulation of the IFN-I pathway by FFV PEVs was detected only in the highly metastatic MV3 spheroids. In melanoma, interferon signaling pathways are usually downregulated which is associated with immunotherapy resistance especially in immune checkpoint inhibitor-naïve cells [70]. IFN-Is are generally considered as essential drivers of antitumor immunity, stimulating the ability of the immune cells to eliminate tumor cells. However, it has also been suggested that cancer cells might exploit feedback inhibitory mechanisms of IFN-Is to enhance their own proliferation and survival [71]. Interestingly, also fucoidans have so far been considered to possess anti-inflammatory, anti-cancer, and anti-thrombotic properties [72], whereas our data suggests that FFVs could also induce pro-cancerous properties (by proxy). While multiple platelet receptors have been identified for FFV following its discovery as a platelet agonist [29, 30], our RNA-seq data indicated that the CLEC-2 pathway was activated only by FFV PEVs. In the MV3 spheroids both FFV and CRP PEVs upregulated LGALS3 transcription, which codes for galectin-3.

Differently from FFV PEVs, TGF-β signaling was upregulated only by CRP PEVs in the MV3 spheroids (at 6 h). Given that platelets are capable of regulating inflammation and immunity [9, 73], the composition and effect of CRP and FFV PEVs on different cancer cell types warrant further investigation in the future, e.g. using engineered CRP PEVs and FFV PEVs in drug delivery. Since PEVs are highly tunable by platelet activation, their mechanism of action cannot be fully determined by physicochemical characterization or omics approaches [9], which means that functional assays such as high-throughput drug screening could help to identify affected signaling pathways and combinatorial effects of PEVs on different cancer cells. Inhibiting GPVI and CLEC-2 receptors in mouse melanoma models has been shown to reduce already-established metastases without introducing any bleeding [25, 74, 75]. The cancer-promoting effects of CRP and FFV PEVs found in this study further endorse GPVI and CLEC-2 receptors as promising therapeutic targets once metastatic spread begins.

## Conclusions

This study provides valuable insights into the complex roles of PEVs in cancer such as metastatic melanoma. Common patterns for CRP and FFV PEVs were found in proliferation, invasion, EV production, and the shared modulation of the PI3K-Akt and MAPK signaling pathways relevant for melanoma progression. In contrast, the differential effects of CRP and FFV PEVs on the TGF-β and IFN-I signaling pathways along with altered tetraspanin profiles underscore the complexity of the PEV-mediated immunomodulation, and it is essential to validate their pathological relevance in in vivo animal models. Understanding the molecular interactions between cancer cells and platelets could be advantageous for the development of antiplatelet strategies better aligning with cancer therapy. For example, high-throughput drug screening could be used to uncover altered efficacy in the presence of PEVs. This could help to improve existing treatments, particularly in cases of drug resistance, or to discover novel targets to develop adjuvant drugs in cancer therapy.

## Electronic supplementary material

Below is the link to the electronic supplementary material.

Supplementary Material 1

## Data Availability

All data generated in this study are available upon request. We have submitted all relevant data of our experiments to the EV-TRACK knowledgebase (EV-TRACK ID: EV240159). The RNA-seq data are available in Figshare at https://figshare.com/s/e38dd3f87d0a7847e871.

## Abbreviations

ANOVA: Analysis of variance
BRAF: B-Raf serine-threonine kinase
Ca^2+^: Calcium ionophore
CD: Cluster of differentiation
cDNA: Complementary DNA
CLEC-2: C-type lectin 2 receptor
CRP: Collagen related peptide
CTCs: Circulating tumor cells
CTRL: Control
DEGs: Differentially expressed genes
ECM: Extracellular matrix
ER: Endoplasmic reticulum
EV: Extracellular vesicle
FBS: Fetal bovine serum
FFV: Fucoidan from Fucus vesiculosus
GO: Gene Ontology
GPIbα: Glycoprotein Ib platelet subunit alpha
GPVI: Glycoprotein VI
HUVECs: Human umbilical vein endothelial cells
IFN-I: Type-I interferon
KEGG: Kyoto Encyclopedia of Genes and Genomes
MAPK: Mitogen-activated protein kinase
MMP: Matrix metalloproteinase
MEK: Mitogen-activated protein kinase kinase
NFC: Nanofibrillar cellulose
NTA: Nanoparticle tracking analysis
PAI-1: Plasminogen activator inhibitor 1
PD-L1: Programmed death-ligand 1
PEAR1: Platelet endothelial aggregation receptor 1
PEV: Platelet-derived extracellular vesicle
PI3K-Akt: Phosphotylinosital 3 kinase - protein kinase B
PMEPA1: Prostate transmembrane protein, androgen induced 1
RNA-seq: RNA-sequencing
RT: Room temperature
RT-qPCR: Quantitative reverse transcription PCR
SERPINE1: Serine protease inhibitor family E member 1
SP-IRIS: Single particle interferometric reflectance imaging sensing
TC: Thrombin & collagen
TGF-β: Transforming growth factor-beta
