# Correction: Platelet-derived extracellular vesicles induced through different activation pathways drive melanoma progression by functional and transcriptional changes

**DOI:** 10.1186/s12964-024-02026-6

**Published:** 2025-01-07

**Authors:** Zeynep Tavukcuoglu, Umar Butt, Alessandra V. S. Faria, Johannes Oesterreicher, Wolfgang Holnthoner, Saara Laitinen, Mari Palviainen, Pia R-M Siljander

**Affiliations:** 1 EV group, Molecular and Integrative Biosciences Research Programme, Faculty of Biological and Environmental Sciences, and CURED, Drug Research Program, Faculty of Pharmacy, Division of Pharmaceutical Biosciences, University of Helsinki, Viikinkaari 9, Helsinki, 00790 Finland; 2 Faculdade Israelita de Ciências da Saúde Albert Einstein, Hospital Israelita Albert Einstein, São Paulo, SP Brazil; 3 AUVA Research Centre, Ludwig Boltzmann Institute for Traumatology, Vienna, Austria; 4 Finnish Red Cross Blood Service (FRCBS), Helsinki, Finland; 5 EV Core, Molecular and Integrative Biosciences Research Programme, Faculty of Biological and Environmental Sciences, University of Helsinki, Helsinki, Finland

**Correction: Cell Commun Signal 22**,** 601 (2024)**

10.1186/s12964-024-01973-4

Following publication of the original article [1], the authors identified a typesetting error in the author name Alessandra V. S. Faria.

The incorrect author name is: Alessandra V. Sousa de Faria.

The correct author name is: Alessandra V. S. Faria.

The publishers apologize for the error. The author group has been updated above and the original article [1] has been corrected.
