# Supplementary material for: Platelet-derived extracellular vesicles induced through different activation pathways drive melanoma progression by functional and transcriptional changes
Source: Cell Commun Signal. 2024 Dec 18;22:601. doi: 10.1186/s12964-024-01973-4 (PMC11658145; doi:10.1186/s12964-024-01973-4)
Supplement: Supplementary file 1 — Supplementary Material 1 [file 12964_2024_1973_MOESM1_ESM.docx]

# Supplementary Tables and Figures

**Supplementary Table 1.** MV3 and A2058 cell line characteristics.

| **Cell line** | **MV3** | **A2058** | **References** |
| --- | --- | --- | --- |
| Patient | 76-years-old male | 43-years-old male | (1) |
| Organ | Skin | Skin | (1) |
| Disease | Amelanotic melanoma | Amelanotic melanoma | (1) |
| Morphology | Epithelial | Epithelial | (1) |
| Derived from | Lymph node | Lymph node | (1) |
| MSI status^*^ | NA | MSS^**^ | (1) |
| *TP53* | NA | p.Val274Phe (c.820G>T) | (1,2) |
| *BRAF* | WT | p.Val600Glu (c.1799T>A) | (1–3) |
| *NRAS* | Q61K | WT | (1–3) |
| *TP63* | NA | p.Arg379Cys (c.1135C>T) | (1,2) |
| *TERT* | NA | c.1-124C>T (c.228C>T) | (1,2) |
| *NF1* | NA | Missense | (1,2) |
| *PTEN* | NA | Missense | (1,2) |

*^*^: Microsatellite instability*

*^**:^ Microsatellite stable*

**Supplementary Table 2.** Quantitative reverse transcription PCR (RT-qPCR) primers used to validate RNA-

sequencing (RNA-seq) results.

| **Gene** | **Forward Primer (5’🡪 3’)** | **Reverse Primer (5’🡪 3’)** |
| --- | --- | --- |
| GAPDH | CCGCATCTTCTTTTGCGTCG | GCCCAATACGACCAAATCCGT |
| PMEPA1 | ACGTGCAACTGCAAACGCTC | AAGGACCGTGCAGACAGCTT |
| SERPINE1 | CGAGGTGAACGAGAGTGGCA | TGCCGGACCACAAAGAGGAA |
| TRIM22 | CCGCATAAACGAGGTGGTCA | GAATCTTCTGTCTCTCGATCTGG |
| TGFB1 | GAGCCTGGACACGCAGTACA | TAGTACACGATGGGCAGCGG |
| IFI44 | TGACTGGCCAAGCCGTAGTG | CCTTCTGCCCCATCTAGCCC |
| SMAD6 | CGGGTGAATTCTCAGACGCC | TCGTAGAAGATGCTGACGGC |
| NOX4 | CACAACTGTAACCGCTGCCC | GGAGAGCCAGATGAACAGGCA |


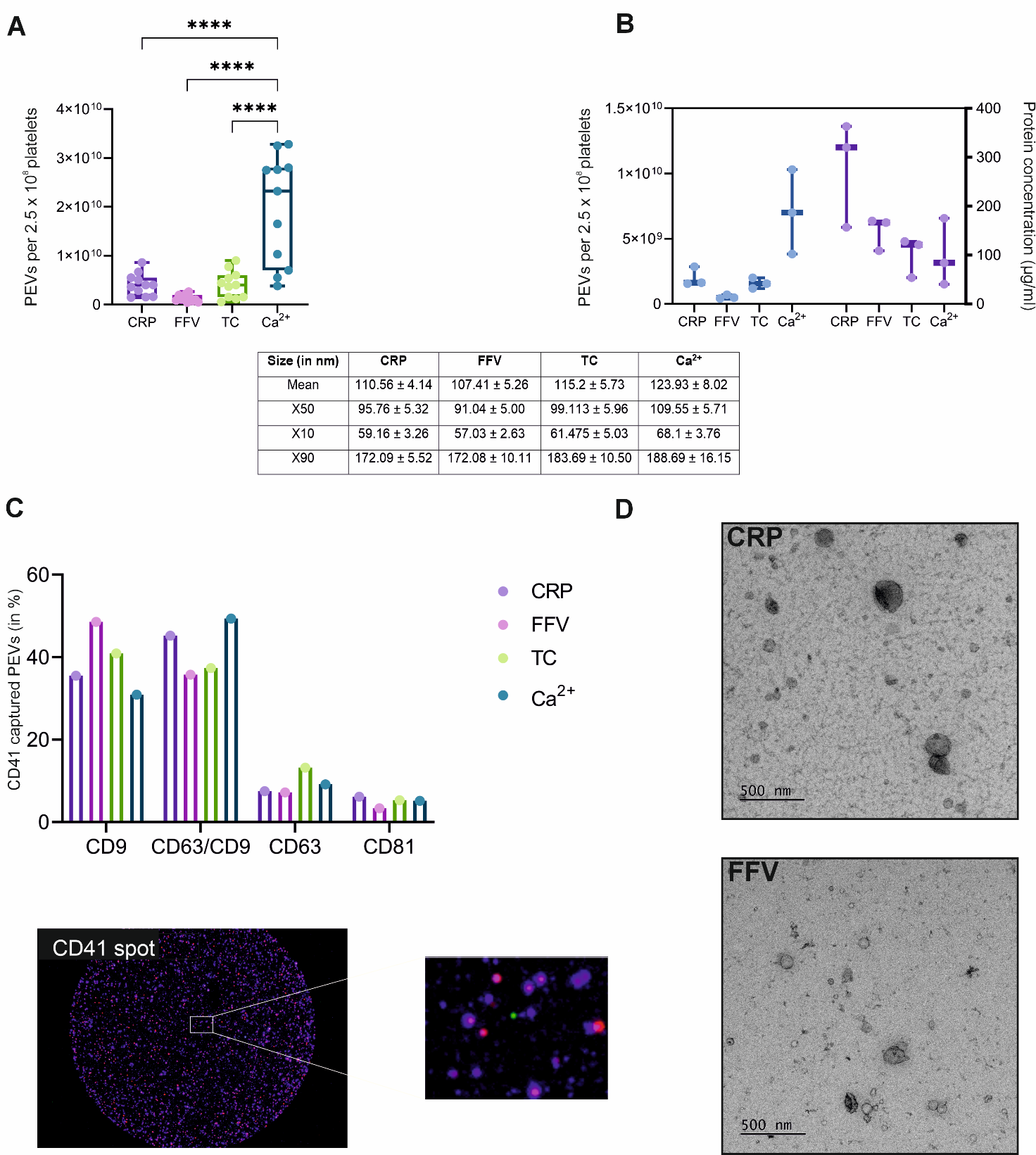


**Fig S1. Characterization of platelet-derived extracellular vesicles (PEVs).** **A** Particle concentration and size distribution of CRP, FFV, TC and Ca^2+^ PEVs were measured by NTA (n = 12; biological replicates representing 48 donors, Statistics: one-way ANOVA followed by Tukey’s multiple comparisons test, * = p < 0.05, ** = p < 0.01, *** = p < 0.001, **** = p < 0.0001). **B** Particle concentration (PEVs per 2.5 x 10^8^ platelets, left axis) and protein concentration (µg/ml, right axis) was analyzed for CRP, FFV, TC, Ca^2+^ PEVs (n = 3; biological replicates representing 12 donors). **C** Tetraspanin profiles (CD63, CD9 and CD81) of the CD41-captured PEVs were measured by SP-IRIS. A representative image of CD41-captured particles labeled with CD9, CD81 and CD63 antibodies is shown. **D** TEM micrographs of negatively stained CRP and FFV PEVs display typical EV morphology.


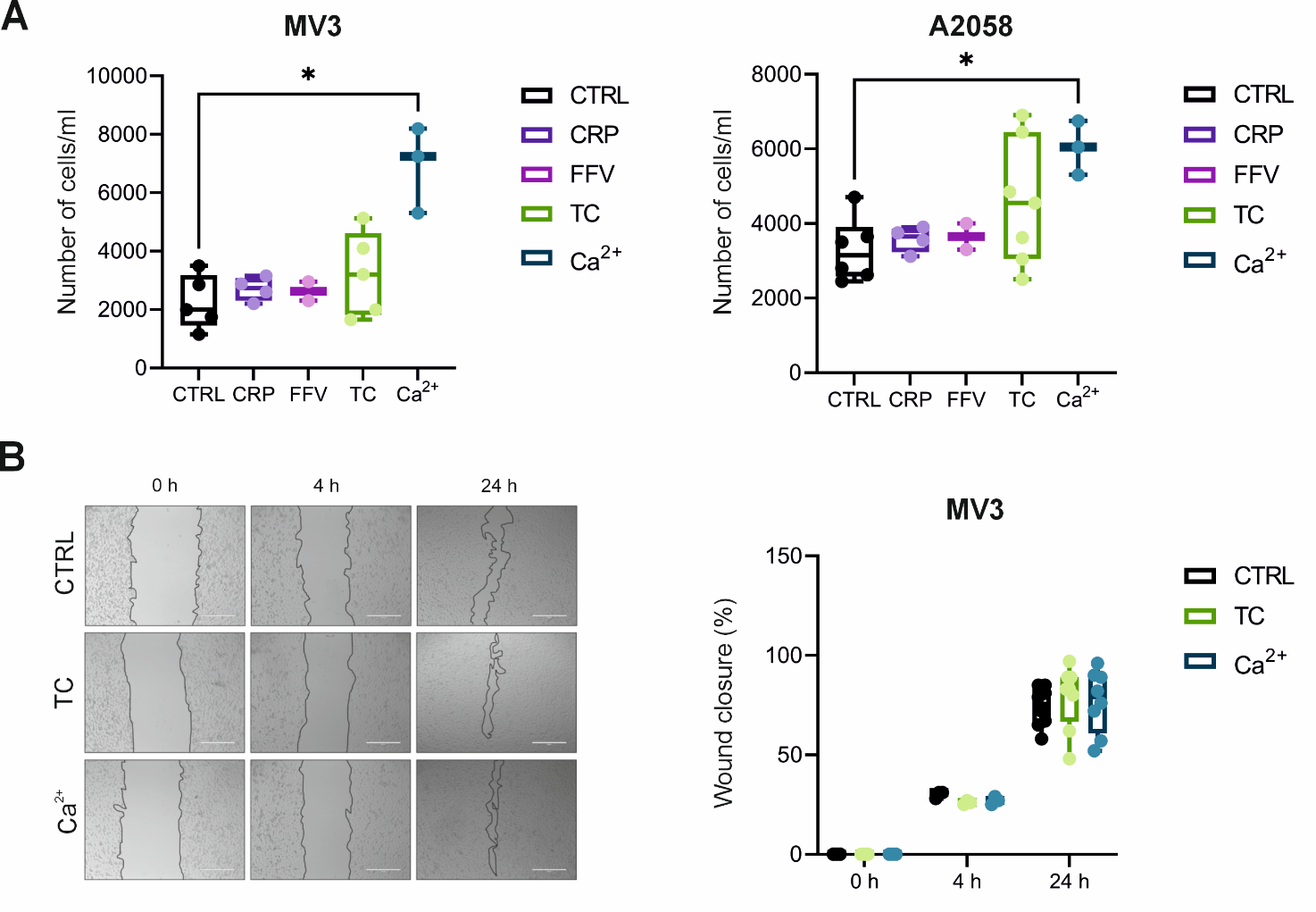


**Fig S2. Cell viability and scratch assay with 2D cultured melanoma cells. A** Cell viability assay (trypan blue exclusion) was conducted with MV3 and A2058 cells that were incubated with 1000 PEVs/cell for 24 h (n = 5 for untreated cells, n = 5; biological replicates representing 20 donors for TC PEVs, n = 4; biological replicates representing 16 donors for CRP PEVs, n = 3; biological replicates representing 12 donors for Ca^2+^ PEVs, n = 2; biological replicates representing 8 donors for FFV PEVs, Statistics: Kruskal-Wallis test, * = p < 0.05) **B** Scratch assay was performed with confluent MV3 cells that were treated with 1000 PEVs/cell for 24 h (n = 6; biological replicates representing 24 donors, Statistics: one-way ANOVA followed by Dunnett’s multiple comparisons test).


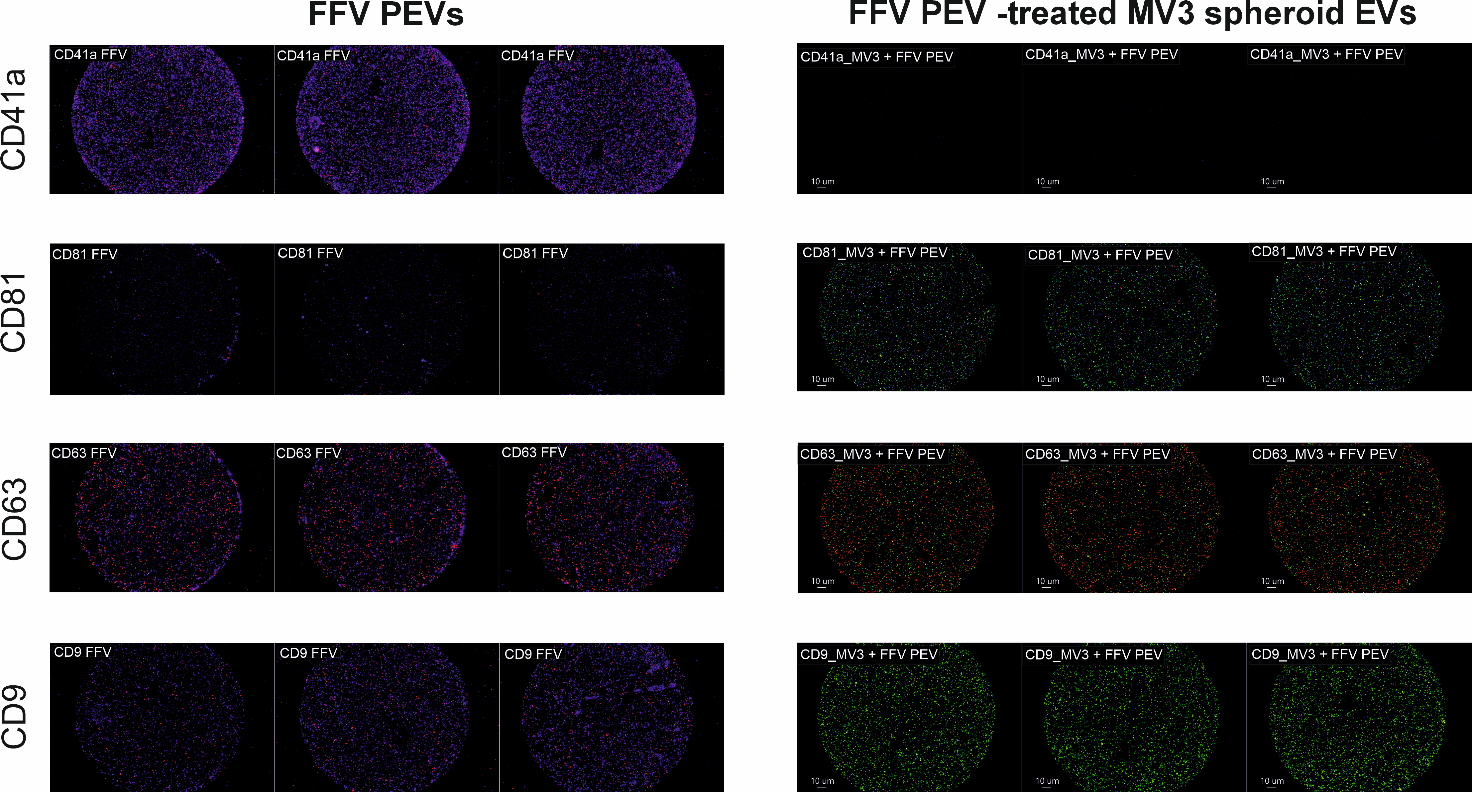


**Fig S3. Fluorescence microscopy images of the CD41a and tetraspanin containing platelet-derived extracellular vesicles (PEVs) and MV3 spheroid-derived EVs analyzed by single particle interferometric reflectance imaging sensing (SP-IRIS).** EVs containing platelet-specific CD41a, and EV tetraspanins (CD9, CD63, CD81) were targeted by capture antibodies. FFV PEVs and FFV PEV -treated MV3 spheroid EVs (3.5 x 10^8^ particles) were loaded onto the chips as measured by NTA (n = 3 technical replicates)**.**


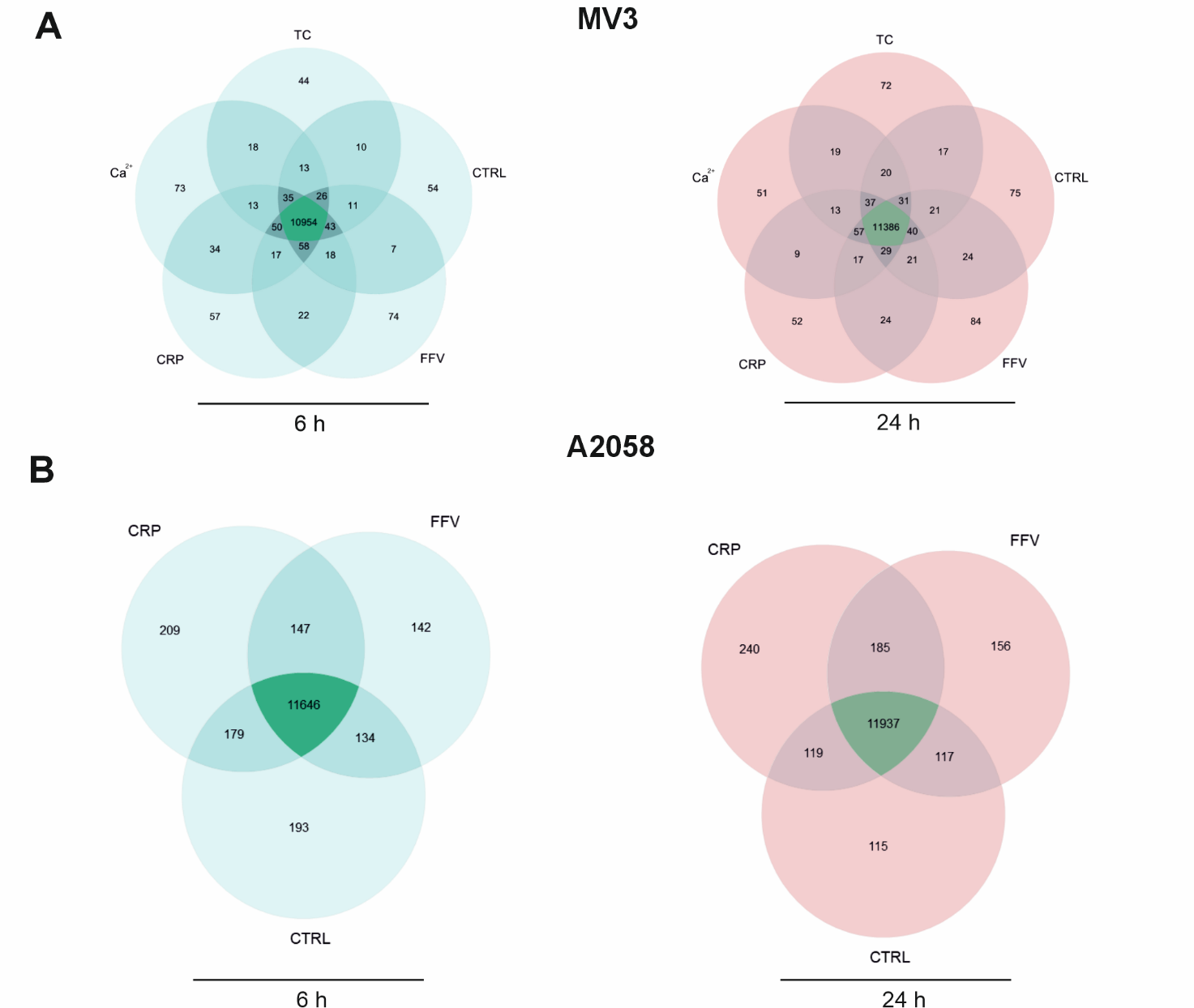


**Fig S4. Commonly and differentially expressed genes between the platelet-derived extracellular vesicle (PEV)-treated and untreated (CTRL) melanoma spheroids. A** Venn diagrams of the CRP, FFV, TC and Ca^2+^ PEV-treated and untreated MV3 spheroids at 6 h and 24 h. **B** Venn diagrams of the CRP and FFV PEV-treated and untreated A2058 spheroids at 6 h and 24 h.


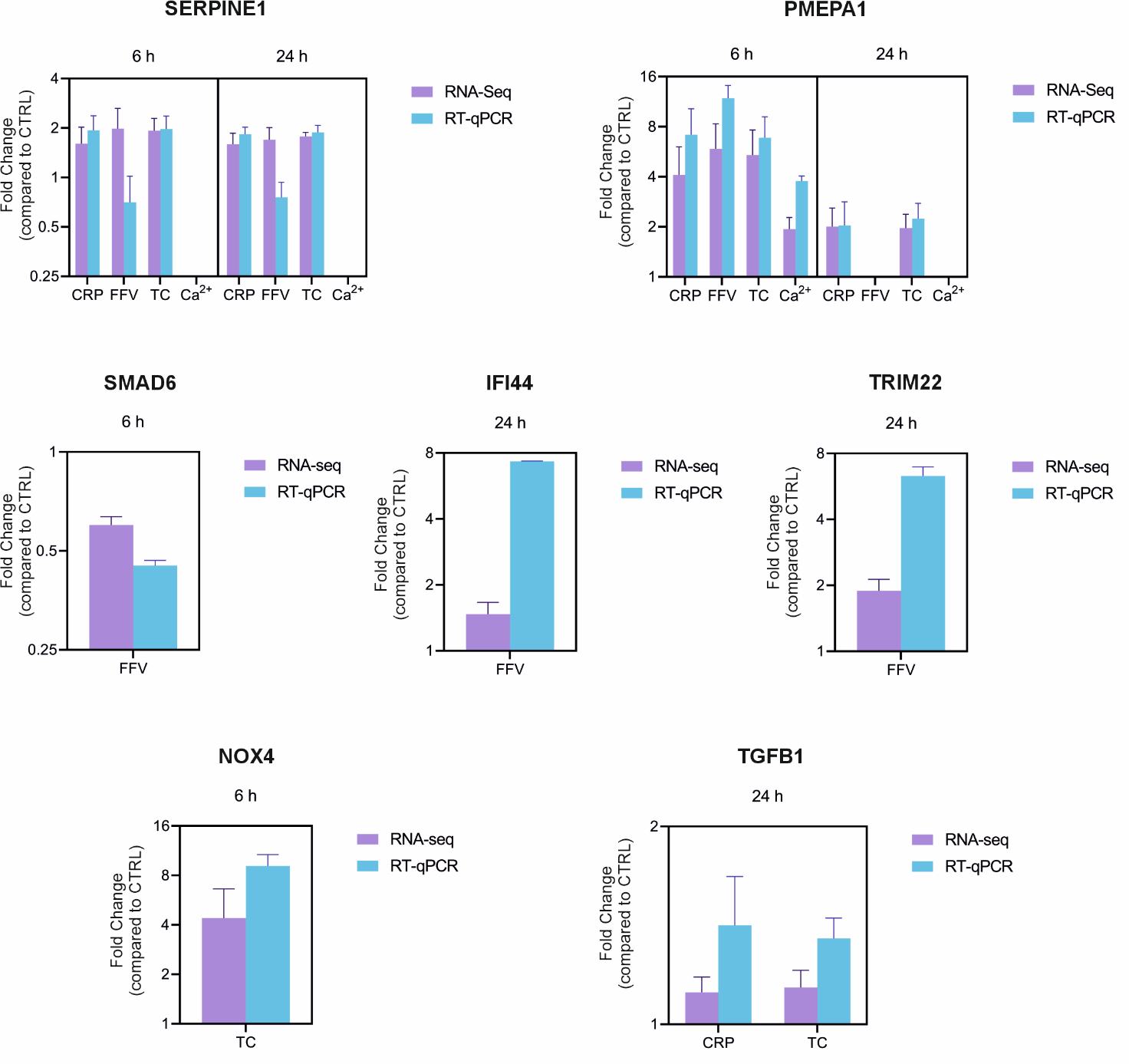


**Fig S5. Validation of RNA-sequencing (RNA-seq) data by quantitative reverse transcription PCR (RT-qPCR).** Fold changes obtained from RNA-seq and RT-qPCR of selected genes in the PEV-treated MV3 spheroids compared to controls at 6 h and 24 h (n = 4; biological replicates representing 16 donors).

**Supplementary Table 3**. Fold changes of the differentially expressed genes SERPINE1, PMEPA1, SMAD6, IFI44, TRIM22, NOX4, TGFB1 in the MV3 spheroids identified by RNA-seq and validated by RT-qPCR.

| **Gene** | **PEV treatment** | **Time (h)^*^** | **RNA-seq^**^**  **)** | **RT-qPCR^**^** |
| --- | --- | --- | --- | --- |
| SERPINE1 | CRP | 6 | 1.61 | 1.94 |
| SERPINE1 | FFV | 6 | 1.98 | 1.67 |
| SERPINE1 | TC | 6 | 1.93 | 1.97 |
| SERPINE1 | CRP | 24 | 1.59 | 1.83 |
| SERPINE1 | FFV | 24 | 1.70 | 0.76 |
| SERPINE1 | TC | 24 | 1.78 | 1.88 |
| PMEPA1 | CRP | 6 | 4.11 | 7.16 |
| PMEPA1 | FFV | 6 | 5.89 | 9.36 |
| PMEPA1 | TC | 6 | 5.41 | 6.87 |
| PMEPA1 | Ca^2+^ | 6 | 1.94 | 3.77 |
| PMEPA1 | CRP | 24 | 2.01 | 1.65 |
| PMEPA1 | TC | 24 | 1.96 | 2.23 |
| SMAD6 | FFV | 6 | 0.60 | 0.45 |
| IFI44 | FFV | 24 | 1.47 | 7.33 |
| TRIM22 | FFV | 24 | 1.89 | 6.31 |
| NOX4 | TC | 6 | 4.45 | 9.13 |
| TGFB1 | CRP | 24 | 1.12 | 1.41 |
| TGFB1 | TC | 24 | 1.14 | 1.35 |

**: Incubation time of the 3^rd^ PEV treatment at day 5*

***: Fold change compared to control*


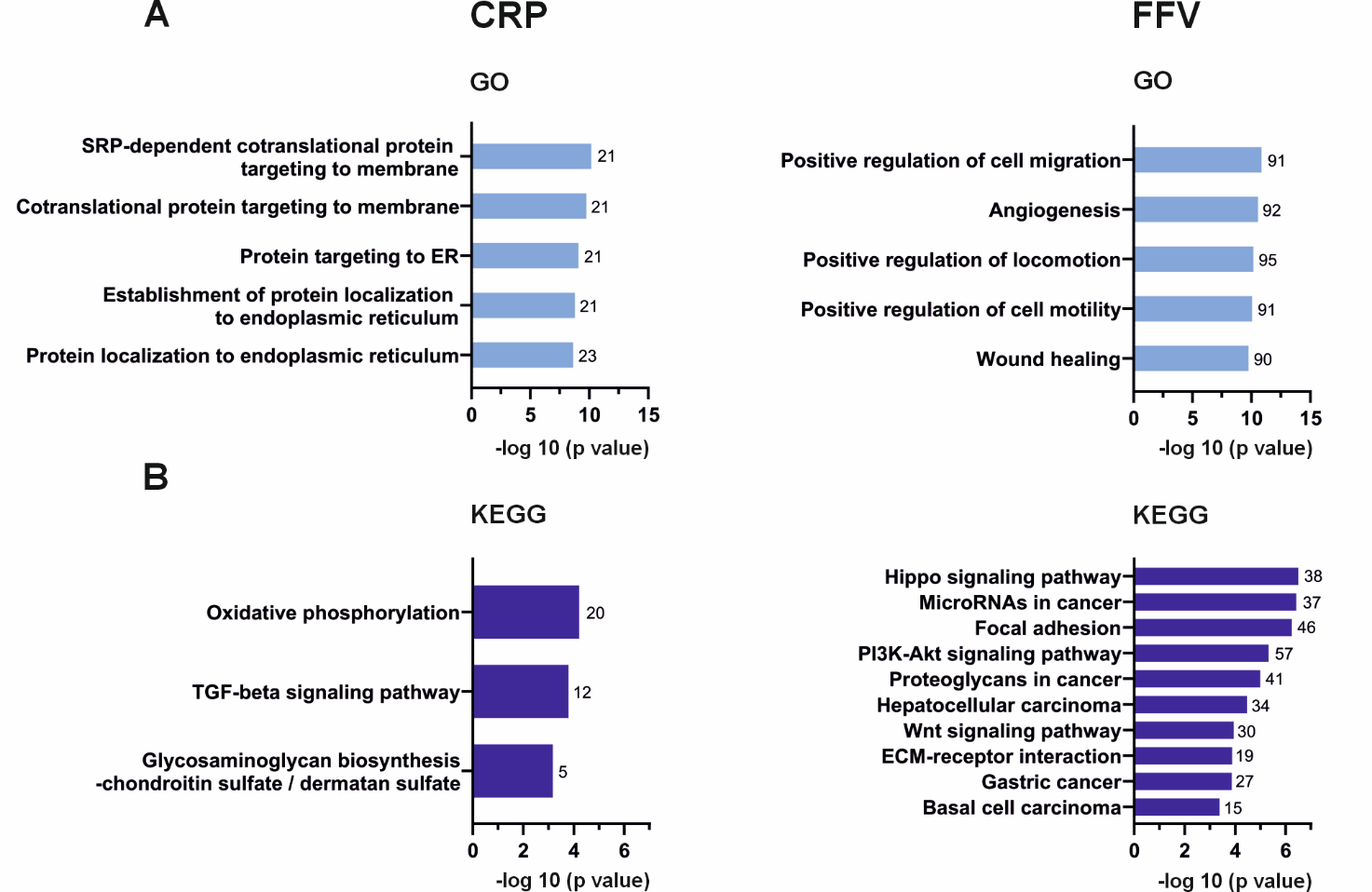


**Fig S6. Gene Ontology (GO) and Kyoto Encyclopedia of Genes and Genomes (KEGG) pathway analyses for the CRP and FFV platelet-derived extracellular vesicle (PEV)-treated MV3 spheroids at 6 h.** On the x-axis, -log10 (p value) is given and on the y-axis, GO or KEGG pathways are given. The number of DEGs that are significantly different (compared to untreated spheroids) is given at the end of the GO term columns. **A** GO biological process analysis showed that CRP PEVs activated more protein targeting and localization, while FFV PEVs activated signaling pathways of cell migration, angiogenesis and wound healing. **B** KEGG analysis showed that CRP PEVs activated oxidative phosphorylation and TGF-β signaling pathway, while FFV PEVs activated Hippo and PI3K-Akt signaling pathway, miRNAs in cancer and focal adhesion (n = 4; biological replicates representing 16 donors, Statistics: Benjamini and Hochberg, adjusted p-value <= 0.05).


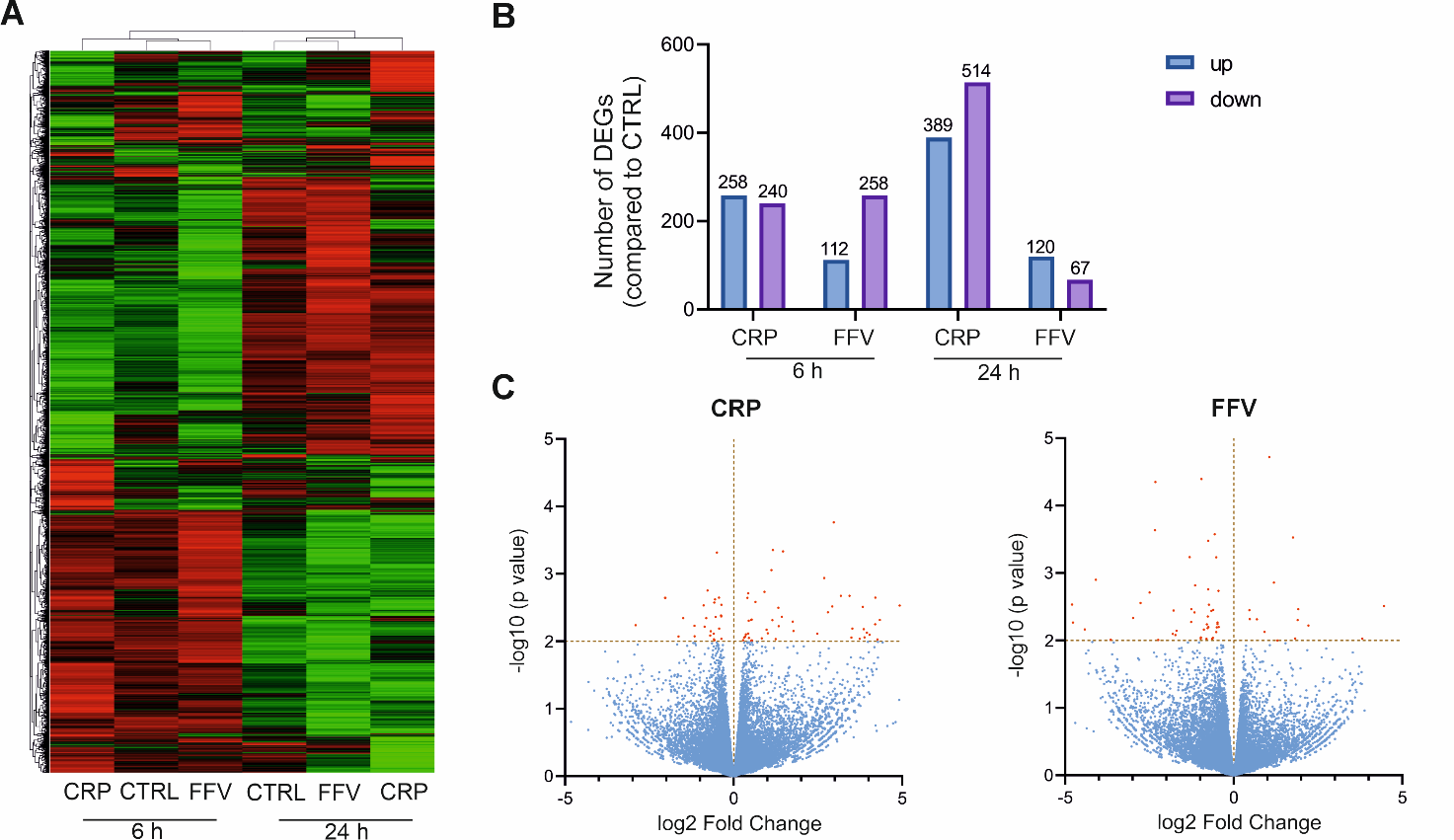


**Fig S7. RNA-sequencing (RNA-seq) of A2058 spheroids treated with CRP and FFV platelet-derived extracellular vesicles (PEVs). A** Hierarchical clustering of the CRP and FFV PEV-treated and untreated A2058 spheroids at 6 h and 24 h. Red color indicates genes with high expression levels, and green color indicates genes with low expression levels. **B** Number of DEGs (up- and downregulated) for the CRP and FFV PEV treatments at 6 h and 24 h. **C** Volcano plots showing the –log10 (p value) vs log2 fold change differences for the CRP and FFV PEV-treated A2058 spheroids compared to untreated spheroids at 6 h. Red dots indicate significant genes while blue dots indicate non-significant genes (n = 4; biological replicates representing 16 donors, Statistics: Benjamini and Hochberg, DESeq2 pvalue<=0.05, |log2FoldChange|>=0.0).


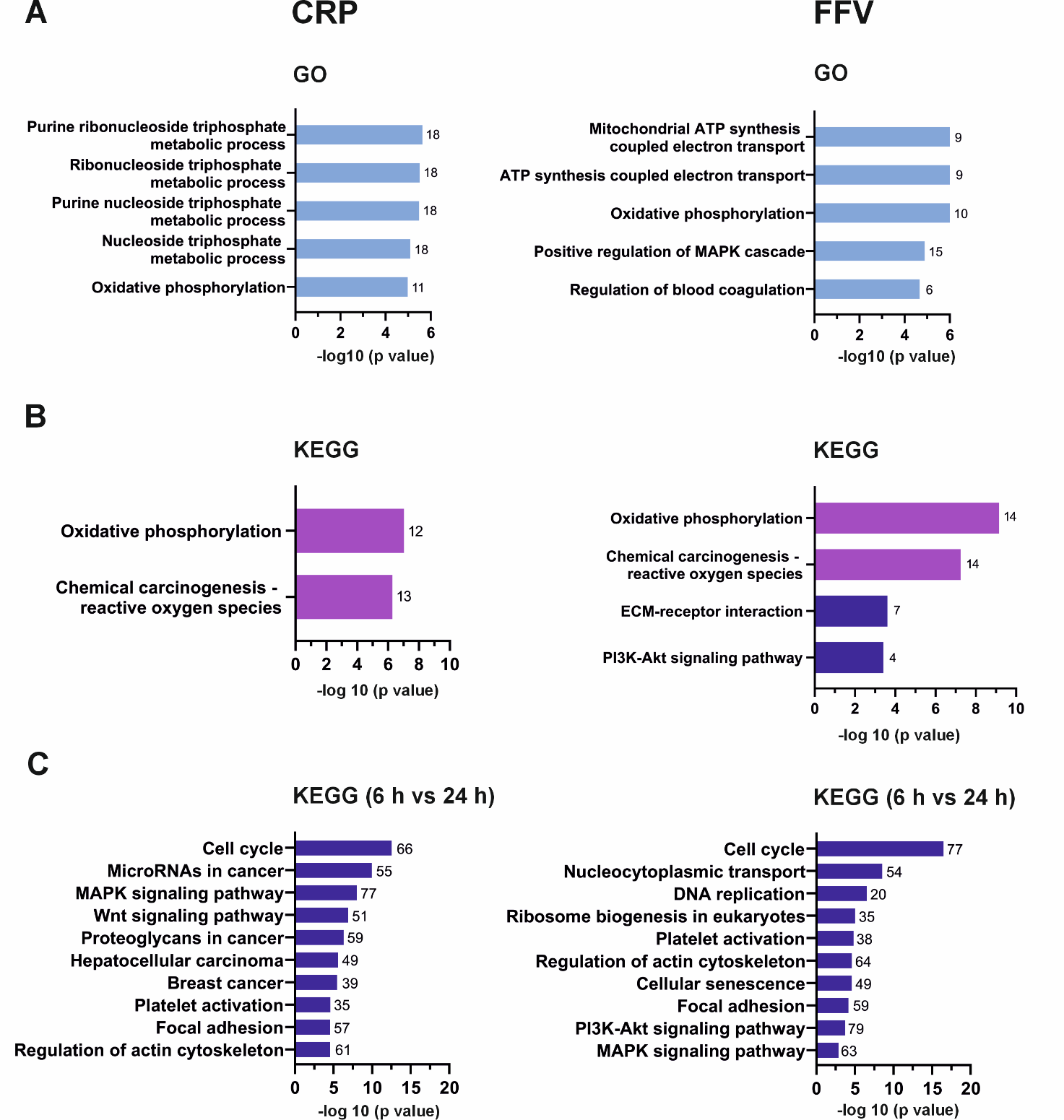


**Fig S8. Gene Ontology (GO) and Kyoto Encyclopedia of Genes and Genomes (KEGG) pathway analyses for the CRP and FFV platelet-derived extracellular vesicle (PEV)-treated A2058 melanoma spheroids.** On the x-axis, -log10 (p value) is given and on the y-axis, GO or KEGG terms are given. **A** GO pathway analysis of the CRP and FFV PEV-treated A2058 spheroids at 6 h. The number of DEGs that are significantly different compared to untreated spheroids is given at the end of the columns for each GO term. **B** KEGG pathway analysis of the CRP and FFV PEV-treated A2058 spheroids at 6 h. The purple color represents downregulated DEGs, whereas the dark blue color represents upregulated DEGs. Oxidative phosphorylation and chemical carcinogenesis-reactive oxygen species were downregulated in the CRP PEV-treated A2058 spheroids. Oxidative phosphorylation and chemical carcinogenesis-reactive oxygen species were downregulated in the FFV PEV-treated A2058 spheroids. ECM-receptor interaction and the PI3K-Akt signaling pathway were upregulated by the FFV PEV -treatment. **C** KEGG pathway analysis of the temporal changes in the CRP and FFV PEV-treated A2058 spheroids. For each pathway, the number of DEGs upregulated at 6 h compared to 24 h is provided for the CRP and FFV PEV -treatments. CRP PEVs significantly upregulated DEGs relevant for cell cycle, microRNAs in cancer, the MAPK signaling pathway, proteoglycans in cancer and platelet activation at 6 h compared to 24 h. FFV PEVs significantly upregulated DEGs in the pathways of cell cycle, platelet activation, regulation of actin cytoskeleton, focal adhesion and the PI3K-Akt signaling pathway at 6 h compared to 24 h (n = 4; biological replicates representing 16 donors, Statistics: Benjamini and Hochberg, adjusted p-value <= 0.05).

## References

1. Duvaud S, Gabella C, Lisacek F, Stockinger H, Ioannidis V, Durinx C. Expasy, the Swiss Bioinformatics Resource Portal, as designed by its users. Nucleic Acids Res. 2021;49(W1):W216–27.

2. Davis EJ, Johnson DB, Sosman JA, Chandra S. Melanoma: What do all the mutations mean? Cancer. 2018;124(17):3490–9.

3. Schrama D, Keller G, Houben R, Ziegler CG, Vetter-Kauczok CS, Ugurel S, et al. BRAFV600E mutations in malignant melanoma are associated with increased expressions of BAALC. J Carcinog. 2008;7:1.
